# Testing the effectiveness of interactive training on sexual harassment and assault in field science

**DOI:** 10.1038/s41598-023-49203-0

**Published:** 2024-01-08

**Authors:** Melissa R. Cronin, Erika S. Zavaleta, Roxanne S. Beltran, Melanie Esparza, Allison R. Payne, Valerie Termini, Joseph Thompson, Megan S. Jones

**Affiliations:** 1 Department of Ecology and Evolutionary Biology, University of California, Santa Cruz, Santa Cruz, CA USA; 2 Nicholas School of the Environment, Duke University, Durham, NC USA; 3 California Department of Fish and Wildlife, Sacramento, CA USA; 4 Los Angeles County Department of Public Health, Los Angeles, CA USA; 5 U.S. Geological Survey, Oregon Cooperative Fish and Wildlife Research Unit, Oregon State University, Corvallis, OR USA

**Keywords:** Human behaviour, Risk factors, Psychology and behaviour

## Abstract

**Abstract:**

Fieldwork is a critical tool for scientific research, particularly in applied disciplines. Yet fieldwork is often unsafe, especially for members of historically marginalized groups and people whose presence in scientific spaces threatens traditional hierarchies of power, authority, and legitimacy. Research is needed to identify interventions that prevent sexual harassment and assault from occurring in the first place. We conducted a quasi-experiment assessing the impacts of a 90-min interactive training on field-based staff in a United States state government agency. We hypothesized that the knowledge-based interventions, social modeling, and mastery experiences included in the training would increase participants’ sexual harassment and assault prevention knowledge, self-efficacy, behavioural intention, and behaviour after the training compared to a control group of their peers. Treatment–control and pre-post training survey data indicate that the training increased participants’ sexual harassment and assault prevention knowledge and prevention self-efficacy, and, to a lesser extent, behavioural intention. These increases persisted several months after the training for knowledge and self-efficacy. While we did not detect differences in the effect of the training for different groups, interestingly, post-hoc tests indicated that women and members of underrepresented racial groups generally scored lower compared to male and white respondents, suggesting that these groups self-assess their own capabilities differently. Finally, participants’ likelihood to report incidents increased after the training but institutional reports remained low, emphasizing the importance of efforts to transform reporting systems and develop better methods to measure bystander actions. These results support the utility of a peer-led interactive intervention for improving workplace culture and safety in scientific fieldwork settings.

**Protocol registration:**

"The stage 1 protocol for this Registered Report was accepted in principle on August 24, 2022. The protocol, as accepted by the journal, can be found at: 10.6084/m9.figshare.21770165.

## Introduction

Fieldwork is a critical tool for scientific research, particularly in applied natural and social science disciplines, including those integral to addressing urgent problems such as climate change^1,2^. Scientific fieldwork, broadly understood as research or data collection conducted outside of a conventional office or laboratory environment^3^, is also central to ecosystem management efforts by government agencies and nonprofit organizations, including monitoring of threatened or invasive species, assessments of habitat quality, and hands-on management efforts such as wildfire fighting. However, fieldwork is often unsafe, particularly for members of historically marginalized groups and those whose presence in scientific spaces threatens traditional hierarchies of power and authority^4–6^. Much attention has been paid in particular to the overt and subtle ways that women, gender minorities, racially marginalized groups, and other underrepresented individuals have been made to feel unwelcome and unsafe in scientific spaces generally^7^ and in field settings specifically^8^. Often these threats are interconnected, with racially and sexually marginalized groups such as women of color reporting higher rates of abuse at work and in science^9–11^.

Sexual harassment—sometimes referred to as sex-based or gender-based harassment because it is rarely sexually motivated^12–14^—and sexual assault are widely documented, as are the negative consequences on victims’ well-being and careers^15,16^. Research has also identified negative repercussions for the workplace as a whole, such as studies linking masculinity contest cultures (i.e., work environments that reward strength, overconfidence, and competitiveness) to lower psychological safety^17^ and organizational performance^18^. Sexual harassment can also negatively impact physical and mental health^19,20^, and the lasting mental and physical consequences of sexual assault are well-documented^21–23^.

Expanding field safety requires minimizing the risk of harassment, assault, and bullying. Addressing these concerns is important for employers and academic organizations that are legally and morally bound to protect their students and staff in the field. Effective action requires research to identify which types of interventions work to prevent field-based sexual harassment and assault from occurring in the first place. Prevention can be understood as a wider and more productive lens than response, as prevention seeks to protect field participants from becoming victimized in the first place. Prevention includes precautionary measures such as the creation of substantive policies^8^, systematic attention to positive organizational culture^17^, and meaningful training of participants and staff in how to model best practices for field safety^24^. These trainings may include recommendations for how to respond effectively to incidents of harassment and assault including establishing confidential reporting channels, sanctioning of perpetrators, and protection of victims and reporters from retaliation. Beyond simply preventing risk, these trainings and recommendations may be doubly beneficial because they can send an early signal to establish a field culture that discourages future hostile behaviour^10^. While prevention trainings are becoming more commonplace in traditional academic and policy settings, few prevention initiatives exist to specifically target the unique high-risk setting of fieldwork^25^.

An important target for harassment prevention programs is the perceived ability to execute desired behaviours, termed self-efficacy^24,26^. Training programs that incorporate a bystander intervention approach (i.e. active helping by third parties who observe an incident) have been associated with greater self-efficacy^27,28^ which in turn has been associated with actual behaviours^27,29,30^. Beyond individual self-efficacy, a second important goal for harassment prevention programs is collective efficacy, or the perceived ability of a community to execute desired behaviours^31,32^. Finally, increasing knowledge of sexual harassment and assault definitions and resources is a baseline goal for most prevention training programs^33,34^.

This study documents a collaboration between scientists at academic institutions and decision-makers at a government agency to administer and evaluate a sexual harassment and assault prevention training program (known as Building a Better Fieldwork Future training, or BBFF training) led by trained agency staff. We used the agency rolling out this training to several hundred staff as a quasi-experiment: participants were not randomly assigned, but we compared before/after changes within subjects and used staff who had not yet been trained as a control. The training itself included knowledge-based interventions, social modeling, and mastery experiences, all of which have been linked to greater self-efficacy elsewhere^35–37^ but which have yet to be studied in sexual harassment and assault prevention. Our research questions [RQs] are:

1. Does participation in the training increase participants’ capacity to take action to create inclusive, safe field environments?
  Hypothesis H1a: Post-training prevention self-efficacy and knowledge will increase significantly in the intervention group compared to the control.
  Hypothesis H1b: Changes in primary outcomes will not be sustained over time.
2. Does participation in the training increase participants’ actions to create inclusive, safe field environments?
  Hypothesis H2a: Post-training prevention behavioural intention will increase significantly in the intervention group compared to the control.
  Hypothesis H2b: Changes in primary outcomes will not be sustained over time.
3. Does the training work equally well for all demographic groups?
  Hypothesis H3a: Increases in post-training knowledge, self-efficacy, and behavioural intention will be higher for women and gender minorities compared to men.
  Hypothesis H3b: No significant differences will be observed in post-training outcomes based on gender, age, race/ethnicity, role, region, or time at the agency.
  Hypothesis H3c: Increases in post-training behavioural intention and self-reported behaviour will be higher for staff who reported higher levels of pre-training prevention behaviour and prevention personal norms compared to their less engaged and committed peers.
4. Do reporting rates increase after participants receive information about sexual harassment and assault?
  Hypothesis H4a: Post-training confidence in reporting and likelihood to report an incident of sexual harassment and assault will be higher in post-training surveys than pre-training.

In addition to these core research questions, we also determined the baseline level of participation in and support for sexual harassment and assault prevention actions at the agency.

## Methods

### Pilot data

A pilot study assessing the efficacy of the BBFF harassment prevention program involved the distribution of surveys to participants prior to and after completing the training. These surveys asked participants to rate their degree of agreement on a Likert scale with the following four statements, which were informed by previous evaluative research^29,38,39^ on self-efficacy and knowledge outcomes of sexual harassment and assault bystander intervention training:

1. I feel knowledgeable about existing resources to help me prevent, intervene in, and report sexual harassment and assault in field settings;
2. I feel confident in my ability to prevent sexual harassment and assault in field settings;
3. I feel confident in my ability to intervene in an incident of sexual harassment and assault in a field setting; and
4. I feel confident in my ability to report an incident of sexual harassment and assault in a field setting.

Surveys were collected immediately prior to and after participating in the BBFF training from 2019 to 2021. Paired pre-post results demonstrated that participants (n = 181) reported significantly (1) greater knowledge about resources to prevent sexual harassment and assault in field settings; and greater confidence in (2) preparing for, (3) intervening in, and (4) reporting sexual harassment and assault in scientific fieldwork settings after completing the BBFF training (Wilcoxon signed-rank tests, p < 0.001, Cronin et al., unpublished data). This pilot study was conducted using mainly academic scientists and practitioners conducting fieldwork, including researchers, university faculty, and graduate and undergraduate students. This narrow study examined a small pool of respondents and only asked four questions related to prevention knowledge and self-efficacy. Still, results suggested that the BBFF training has benefits for self-reported measures and provides the foundation for the deeper analysis described here.

### Study design

We ran a pre-post, non-randomized intervention study of a training delivered to staff at the California Department of Fish and Wildlife (CDFW). CDFW is a large state agency responsible for managing and protecting the state's wild plants, animals and ecosystems. As part of this mission, CDFW requires employees across its seven regions in the state to engage in substantial fieldwork. We used both between-subjects and within-subjects comparisons to determine impact of the training on participants. Training participants were CDFW employees who participate in or manage field science and research. This includes scientific aides, who collect fishery data and biological samples at a range of field sites, the direct supervisors of scientific aides, and the regional managers of each of the seven regions. All staff had previously received mandatory sexual harassment prevention training (which is not specific to fieldwork settings) in relevant CDFW policies and reporting protocols.

A small group (17) of CDFW staff who were nominated by their supervisors from each region were trained by the BBFF Program to become Certified Instructors via a “train the trainers” session consisting of a two-day intensive virtual workshop where instructors received information on content, facilitation, and common questions, followed by a practice session during which instructors delivered a mock training to BBFF staff. These instructors then delivered the same 90-min training to CDFW staff in their region. Trainings for CDFW staff were supervised by experienced BBFF staff.

Each training followed a predetermined script with the same content and lasted 90 min. The training had five major components: (1) introduction to harassment and assault in fieldwork, (2) preparation, (3) intervention, (4) reporting, and (5) scenario-based discussions. In, the facilitator explained why fieldwork settings are particularly high-risk for sexual harassment and assault and outlined the legal and institutional definitions of harassment and assault. In (preparation), the facilitator shared best practices for field-ready protocols, including Codes of Conduct, community agreements, Field Safety plans, and privacy, medical, and other protocols. In (intervention), participants learned basic bystander intervention tools. In (reporting), the facilitator explained the importance of reporting, reporting requirements for staff, and how the reporting process works at CDFW. The scenario-based discussions were interspersed throughout the training, each related to the information and skills learned in the section prior, and range in severity from instances of gender bias to assault in field settings (Box 1). Participants were broken into small groups of three to five members to discuss each scenario for four to five minutes. After discussing in small groups, a larger-group discussion was facilitated by the instructor for five minutes. Trainings were delivered via virtual meeting platform.

The training design incorporated three micro-intervention strategies that have been shown elsewhere to improve self-efficacy: knowledge-based interventions, social modeling, and mastery experiences^31,36,37^. Knowledge-based interventions are designed to increase knowledge about harassment and assault so that participants are more likely to identify it when they observe it^38–40^. Social modeling suggests that people learn to imitate others by replicating their intended and observed behaviours. Finally, mastery experiences are the personal experience of success, whereby participants take on a new challenge and feel that they have succeeded at it, thus building self-efficacy in that area^31^. Components 1–4 of the training constituted the knowledge-based intervention, and social modeling and mastery experiences occurred in the scenario-based discussions.

Survey questions included in this study draw on previously validated survey questions from the Bystander Efficacy Scale, which can be used to assess an individual’s confidence in performing bystander behaviours^27,28^. Survey questions were not replicates of those used in the scale, but gauge confidence about and self-reported likelihood to intervene related to eight main themes as outlined by the scale and similar research on bystander intervention evaluation^26,27,29,30,38,39,41^: knowledge, self-efficacy, personal norms, collective efficacy, self-reported behaviour, behavioural intention, observed behaviour, and training attitude. To adapt our questions to the setting of a government agency, response options were simplified to a seven-point Likert scale rather than the 0–100 used by the original scale (Table S1).

### Sampling design

Staff from each of the seven CDFW regions and the CDFW Office of Spill Prevention and Response were trained from March to June 2022. Each training included 30–40 participants and was led by a certified instructor. Given the restricted nature of field-based employees’ schedules and limited access to reliable WIFI and cell service, random assignment to training sessions was not possible; participants were assigned opportunistically to training sessions based on their availability.

The program was piloted with a single training Southern Coastal California (Region 5), which includes 38 field staff. Trainings were then delivered region by region, in an order determined through discussions with the six other regional managers within CDFW. Trainings were delivered in two batches to create a control group for the quasi-experimental design: the first half of CDFW staff were trained from April 13th to July 5, 2022, and the second half was trained from July 15th until September 25, 2022. At the halfway point post-training data were collected from all staff, allowing for comparison between the intervention group (those who have been trained) and the control group (those who have not yet been trained). Due to constraints associated with staff availability, survey data collection began prior to acceptance of the initial study protocol in the Stage 1 manuscript; however, the data was not accessed before that point, and no data collection protocols were altered after starting data collection.

Surveys were distributed in three waves: Time 1, Time 2, and Time 3, such that treatments are nested within individuals (Table S2). Roughly one week prior to taking the training, participants were invited to complete the Time 1 (pre-training) survey establishing baseline behavioural beliefs, self-reported past behaviour, behavioural intention, and demographics. The invitation email contained a link to the survey platform Qualtrics hosted by UC Santa Cruz. Survey recipients who did not complete the survey received a reminder email roughly two days before their training.

After completing the pre-training baseline survey, participants were contacted by a BBFF Program coordinator to sign up for a 90-min training offered within their region. Immediately after each training, participants were then sent a link to the Time 2 (immediate post-training) survey of behavioural beliefs and intention. Participants received at least one reminder to complete the post-training survey.

At the halfway point (July 6–July 14, 2022), the Time 3 (midpoint) survey midpoint survey data was sent to all staff to measure behavioural beliefs, self-reported past behaviour, and behavioural intention and allow comparison between those who had been trained (treatment group) and those who had yet to be trained (control group). The link to the follow-up survey on Qualtrics was sent via email. Survey recipients received one reminder email a week later. For a full list of predictor, outcome, and control variables measured in each survey wave, see Table S1.

The survey data were supplemented by incident reporting data provided by the CDFW Office of Equal Employment Opportunity's (EEO). After the training program was completed, EEO staff provided aggregated deidentified counts of reported incidents related to sexual harassment from January 2020 to October 2022 (two months after the end of the study period). Reports were coded as either field-based or not, grouped into general categories of incident type (e.g., derogatory comments, hostile work environment, etc.). For reports received after the training program began, EEO staff indicated whether the person who reported the incident was previously trained by the BBFF program. Because incident reports related to sexual harassment are infrequent at CDFW (e.g., fewer than 10 reports per year), these results were not used to test a hypothesis, but were included as anecdotal context in our results.

### Analysis plan

To determine the effect of the trainings on our primary outcomes (RQ1 and RQ2), we ran adjusted and unadjusted linear regressions with post-training knowledge, self-efficacy, prevention behavioural intention and self-reported prevention behaviour (measured 1–2 months after the BBFF training) (Table 1). The preregistered protocol specified that adjusted regressions including demographic variables would match pre-training beliefs and self-reported behaviours; however, due to constraints associated with sample size, only unadjusted regressions could be conducted for treatment–control data. Specifically, we used data from treatment and control groups to determine if treatment condition was a significant predictor of the outcome variable at Time 3 when controlling for that variable at Time 1 (unadjusted regression) or when controlling for that variable and for demographics and the other outcome variables at Time 3 (adjusted regression). We conducted ordinal logistic regressions as a sensitivity analysis, given our outcomes are measured on 7-point scales. The training experience was coded as a binary variable, with the control (not yet trained) as 0 and the treatment (training received) as 1.

**Table 1:** Design table.

Question	Hypothesis	Sampling plan (e.g. power analysis)	Analysis plan	Interpretation given to different outcomes
1. Does participation in the training increase participants’ capacity to take action to create inclusive, safe field environments?	(a) Post-training prevention self-efficacy, collective efficacy and knowledge will increase significantly in the intervention group compared to the control (b) Changes in primary outcomes will not be sustained over time	Our power analysis suggests that with roughly 250 participants in each experimental condition (intervention and control) and assuming a standard deviation of 1 (which is consistent with our pilot data), we are powered to detect an effect size of 0.32 for continuous outcomes with an alpha or 0.05 and power 0.95. Previous research indicates that this is within the range of effect sizes detected in other studies of sexual assault prevention knowledge, self-efficacy and collective efficacy in non-scientific settings^33,44,45^	(a) We will use adjusted and unadjusted linear regressions for responses regarding respondent knowledge, self-efficacy, prevention behavioural intention, and self-reported prevention behaviour (b) We will use multi-level models to compare change in response scores for data nested within an individual from pre-surveys (Time 1) compared to immediately after training (Time 2) and several weeks after training (Time 3)	(a) If the response scores for questions related to self-efficacy, collective efficacy, and knowledge are significantly higher for the intervention group compared to the control (Time 3 survey), we will conclude finding support for Hypothesis 1a (b) If the response scores for questions related to self-efficacy collective efficacy, and knowledge are significantly greater immediately after the training compared to the later time period, we will conclude finding support for Hypothesis 1b
2. Does participation in the training increase participants’ actions to create inclusive, safe field environments?	(a) Post-training prevention behaviour (self-reported) and behavioural intention will increase significantly in the intervention group compared to the control (b) Changes in primary outcomes will not be sustained over time	Same as above	(a) We will use adjusted and unadjusted linear regressions for responses regarding respondent knowledge, self-efficacy, prevention behavioural intention, and self-reported prevention behaviour. We will use repeated-measures ANOVA tests to compare responses at three time points (Times 1, 2, and 3) (b) We will use multi-level models to compare responses for date nested within an individual from pre-surveys (Time 1) compared to immediately after training (Time 2) and several weeks after training (Time 3)	(a) If the response scores for questions related to behaviour (self-reported) and behavioural intention are significantly higher for the intervention group compared to the control, we will conclude finding support for Hypothesis 2a (b) If response changes in scores for questions related to behaviour (self-reported) and behavioural intention are significantly greater immediately after the training compared to the later time period, we will conclude finding support for Hypothesis 2b
3. Does the training work equally well for all demographic groups?	(a) Increases in post-training knowledge, self-efficacy, and behavioural intention and self-reported behaviour will be higher for women and gender minorities compared to men (b) When controlling for gender, no significant differences will be observed in post-training outcomes based on gender, age, race/ethnicity, role, region, or time at the agency (c) Increases in post-training behavioural intention will be higher for staff who reported higher levels of pre-training prevention behaviour and prevention personal norms compared to their less engaged and committed peers	Same as above	(a) We will use linear regressions to compare the effectiveness of the trainings for different groups with CDFW (Times 1 and 2) (b) We will use linear regressions to compare the effectiveness of the trainings for different groups with CDFW controlling for gender (Times 1 and 2) (c) We will use moderation analyses to determine if pre-training prevention behaviour and beliefs moderated the impact of the training on post-training behavioural intention and self-reported behaviour (Times 1 and 2)	(a) If there is significant difference among change in response scores between women and gender minorities and men, we will conclude finding support for Hypothesis 3a (b) If there is no significant difference among change in response scores for participants of different genders, ages, race/ ethnicities, roles, regions, or duration of time at the agency, we will conclude finding support for Hypothesis 3b (c) If there is no significant effect of pre-training behaviour and beliefs on post-training behavioural intention and self-reported behaviour, we will conclude finding support for Hypothesis 3c
4. Do reporting rates increase after participants receive information about sexual harassment and assault?	(a) Post-training confidence in reporting and likelihood to report an incident of sexual harassment and assault will be higher in post-training surveys than pre-training	Same as above	(a) We will use adjusted and unadjusted linear regressions and multilevel models for responses regarding confidence in reporting and likelihood to report. We will use multilevel models repeated-measures ANOVA tests to compare responses at three time points (Times 1,2, and 3)	If the change in response scores (from pre- to post-surveys) for questions related reporting are significantly higher for the intervention group compared to the control and within subjects over time, we will conclude finding support for Hypothesis 4a

We prevented overfitting in our models by checking we have 20 or more observations per variable. If this threshold was not met, we pre-screened potential covariates using a bivariate likelihood ratio test with the outcome, and included covariates with p value less than 0.20. If we found that the training significantly influenced both post-training perceptions (knowledge and self-efficacy) and behavioural measures (behavioural intention and self-reported behaviour) we ran mediation analyses to determine if changes in perception mediated changes in prevention behaviour. Our original registered report design planned for roughly 250 participants in each experimental condition (intervention and control) and assuming a standard deviation of 1, we are powered to detect a difference in means with effect size of 0.32 for continuous outcomes with an alpha of 0.05 and power 0.95. This is within the range of effect sizes detected in other studies of sexual assault prevention knowledge, self-efficacy and collective efficacy in non-scientific settings^33,42,43^. However, due to difficulties obtaining survey responses from participants (see “Study limitations and future research” section in the “Discussion”) our final sample size for treatment–control analyses was 140 participants who completed all three survey waves, 80% (n = 112) of whom were in the treatment group and 20% (n = 28) of whom were in the control group, giving us power to detect an effect size of 0.76 (large effect size) for continuous outcomes with an alpha of 0.05 and power 0.95. Thus, we are underpowered to detect smaller effect sizes for the treatment–control analyses, but remain powered to detect large differences in score changes between the study groups.

We measured longitudinal change within subjects using mixed effects models (also known as multilevel models) to compare responses at three time points for data nested within an individual (*RQ1, RQ2,* Times 1, 2, and 3)^44^. We used these models to compare the effectiveness of the trainings for different groups with CDFW (RQ3). The preregistered protocol specified that we would run additional ANOVA comparisons; however it was determined that multilevel models were more appropriate to test for differences between time points for the data. Using within-subjects comparisons, we looked for differences in training outcomes by gender, race/ethnicity, age, seniority (years employed at CDFW and position within the agency), region, and duration of time employed at CDFW. For race/ethnicity analyses, demographic data were scored as binomial (Yes = 1, No = 0) for under-represented minority (URM) status for respondents who identified primarily as African American/Black, American Indian/Alaskan Native, and/or Hispanic/Latino. Respondents who chose not to specify demographic data were not included in these analyses. We ran moderation analyses to determine if pre-training prevention behaviour and beliefs moderated the impact of the training on post-training behavioural intention and self-reported behaviour.

The preregistered protocol specified that we would run tests for collective efficacy and prevention behaviour (self-reported) using treatment–control data (Table 1); however, our Time 1 and Time 3 survey questions did not adequately measure these variables (Table S1); thus these tests were omitted.

### Exploratory analyses

Given sample size limitations for treatment–control data that prevented the use of adjusted regressions, we used the larger within-subjects dataset to conduct adjusted regressions for the effects of race and gender of change between pre- and post-training scores related to knowledge, self-efficacy, and behavioural intention.

### Ethical approval

The research complied with all relevant ethical regulations, and a University of California (UC) Santa Cruz Internal Review Board (IRB) permit was received for research involving human subjects (UCSC #HS-FY2022-226). Informed consent was sought from all participants prior to data collection.

## Results

### Sample groups

A total of 44 trainings were delivered to 925 CDFW employees between April and August 2022. After filtering out for empty and duplicate surveys, a total of 1048 surveys representing 630 participants were completed across the three waves (Table S3). We used three datasets to conduct analyses: (1) for treatment–control comparisons, we matched 140 participants who completed both the pre-training survey (Time 1) prior to the midpoint, and also completed the midpoint survey (Time 3), 80% (n = 112) of whom were in the treatment group and 20% of whom were in the control (n = 28). (2) For longitudinal analyses using multilevel models, we matched 64 individuals who completed all three survey waves *and* were in the treatment group for the Time 3 survey (so that the immediate post-training survey and 1–2 months post-training surveys were both completed after receiving the training). (3) For within-subjects pre- and post-training comparisons, we matched 196 participants who completed both pre-training (Time 1) and post-training (Time 2) surveys (Tables S2, S3); of these, surveys that included responses for gender (n = 173) and race (n = 165) were used for exploratory analyses.

For questions that were asked separately for harassment and assault, we grouped responses for highly correlated responses with Cronbach’s alpha > 0.8 into a single mean score (Fig. S1).

#### Does participation in the training increase participants’ capacity to take action to create inclusive, safe field environments?

*H1a. Post-training prevention self-efficacy and knowledge will increase significantly in the intervention group compared to the control.*

**Figure 1:**
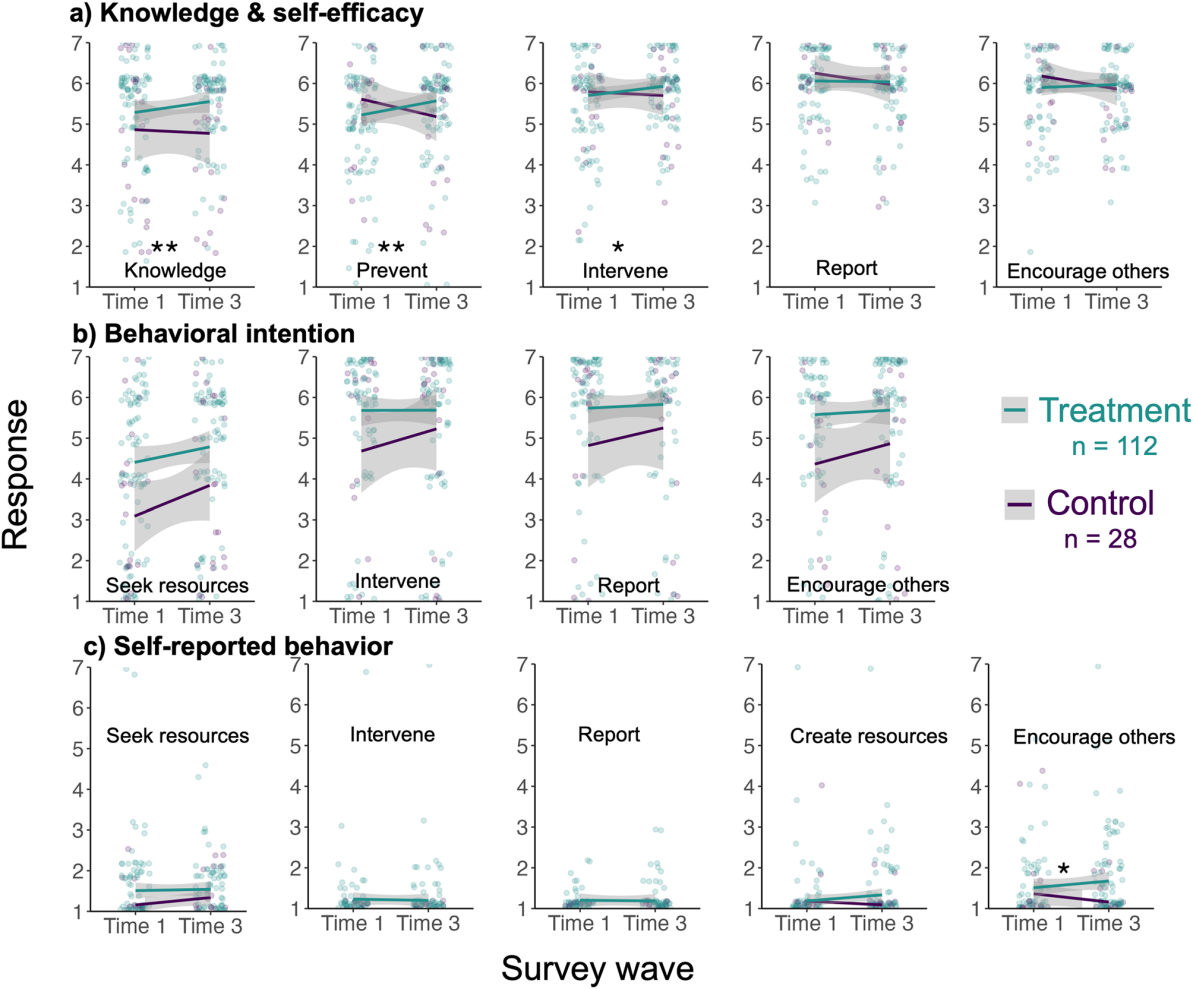
Linear model results for treatment and control group responses for survey questions for sexual harassment and assault related to (**a**) knowledge and self-efficacy, (**b**) behavioural intention, and (**c**) self-reported behaviour at two time points: pre-training (Time 1) and 1–2 months post-training for treatment and control groups (Time 3) (n = 140).

Linear regression results indicate significant increases in the treatment group compared to the control group in self-reported knowledge and prevention and intervention self-efficacy (p < 0.05, Table 2, Fig. 1a) The largest effect sizes were for knowledge (β = 0.84, 95% CI [0.30, 1.38], p = 0.003) and prevention self-efficacy (β = 0.74, 95% CI [0.24, 1.23], p = 0.004). We did not find significant differences between the treatment and control groups in the change in the other two forms of self-efficacy (reporting and encouraging others, Table 2, Fig. 1a), indicating partial support for Hypothesis 1a.

*H1b. Changes in knowledge and self-efficacy will not be sustained over time*.

**Table 2:** Linear model results for treatment (n = 112) and control group (n = 28) responses for survey questions for sexual harassment and assault related to knowledge and self-efficacy, and behavioural intention at two time points: pre-training (Time 1) and 1–2 months post-training (Time 3) (n = 140).

Concept	Variable	β	CI lower	CI upper	Std. error	p value	Sig
Knowledge	Knowledge	0.839	0.296	1.381	0.274	0.003	**
Self-efficacy	Preventing	0.736	0.242	1.230	0.249	0.004	**
	Intervening	0.437	0.061	0.812	0.190	0.023	*
	Reporting	0.140	− 0.303	0.583	0.224	0.534	
	Encouraging others	0.369	− 0.042	0.779	0.207	0.078	
Behavioural intention	Seeking resources	0.487	− 0.353	1.327	0.424	0.253	
	Intervening	0.252	− 0.628	1.131	0.444	0.572	
	Reporting	0.292	− 0.572	1.155	0.436	0.505	
	Encouraging others	0.375	− 0.458	1.209	0.421	0.375	
Self-reported behaviour	Seeking resources	0.094	− 0.290	0.478	0.194	0.629	
	Intervening	0.171	− 0.122	0.464	0.148	0.250	
	Reporting	0.143	− 0.149	0.436	0.148	0.334	
	Creating resources	0.230	− 0.117	0.578	0.176	0.192	
	Encouraging others	0.493	0.038	0.947	0.229	0.034	*

Asterisk denote statistically significant p value (*p < 0.05; **p < 0.01; ***p < 0.001).

**Figure 2:**
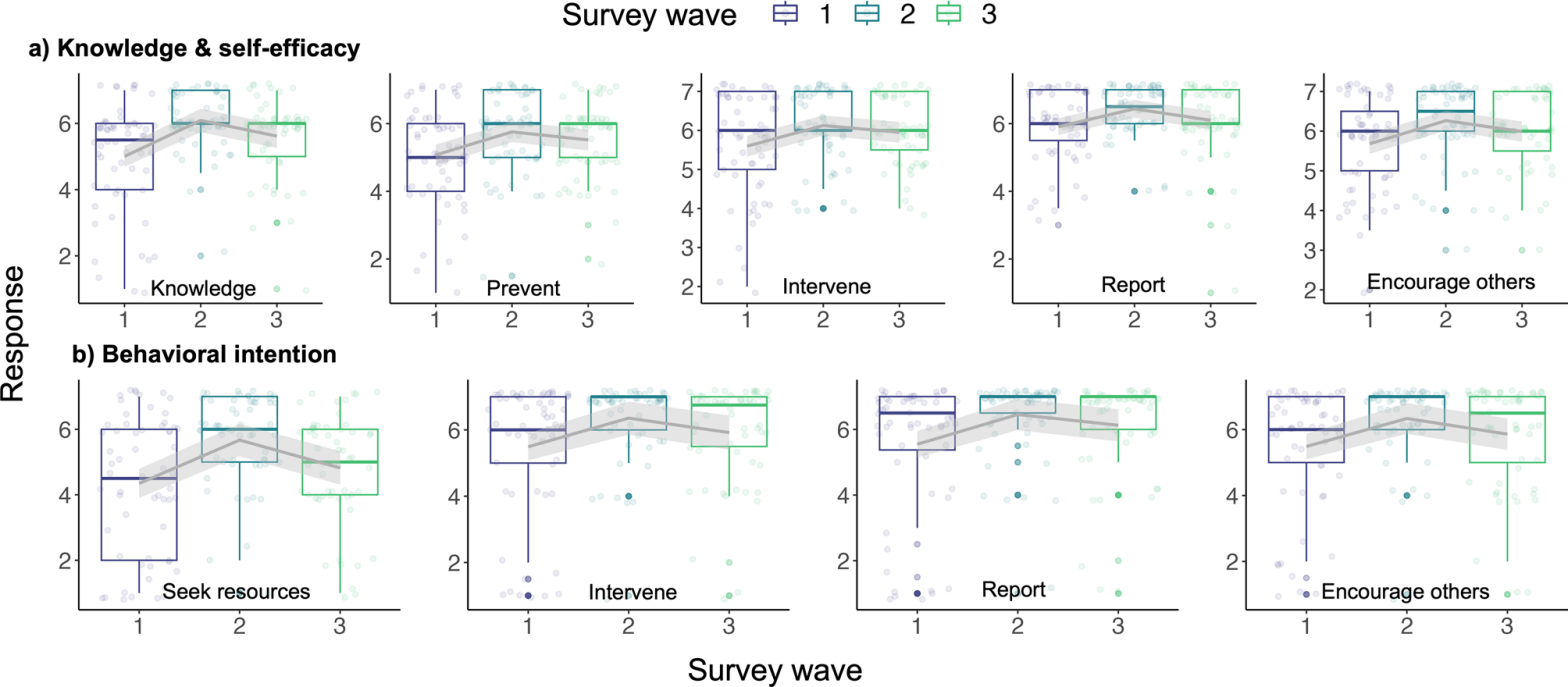
Longitudinal multilevel models showing survey responses at three time points within-subjects: pre-training (Time 1), immediately post-training (Time 2), and 1–2 months post-training (Time 3) for survey questions related to (**a**) knowledge and self-efficacy and (**b**) behavioural intention (n = 64).

Within-subjects comparisons revealed significant increases in self-reported knowledge (β = 1.1 95% CI [0.75–1.45], p < 0.001) and all forms of self-efficacy immediately after the training (β = 0.53–0.69, 95% CI [range 0.26–0.99], p < 0.001, Table 3, Fig. 2a). This effect was sustained in our within-subjects comparison 1–2 months after the training delivery for self-reported knowledge (β = 0.61, 95% CI [0.24, 0.97], p < 0.001) and prevention, intervention, and encouragement self-efficacy (β = 0.18–0.45, 95% CI [range 0.05–0.76], p < 0.01). We did detect a drop-off in the intensity of the effect at the 1–2-month mark, but the increase was still significant compared to baseline (p < 0.01, Table 3). In contrast, for reporting self-efficacy, scores returned to baseline 1–2 months after training delivery. This indicates a rejection of Hypothesis 1b for all variables except reporting self-efficacy.

**Table 3:** Longitudinal multilevel model results (RQ1, Hypothesis H1a and RQ2 Hypothesis H2a) for variables related to knowledge, self-efficacy (RQ1), and behavioural intention (RQ2) concepts between scores pre-training (Time 1), immediately post-training (Time 2), and 1–2 months post-training (Time 3).

Predictors	Knowledge			Preventing			Intervening			Reporting			Encouraging others		
	Estimates	CI	p	Estimates	CI	p	Estimates	CI	p	Estimates	CI	p	Estimates	CI	p
(a) Knowledge and self-efficacy															
(Intercept)	4.98	4.67–5.30	** < 0.001**	5.07	4.76–5.38	** < 0.001**	5.58	5.31–5.85	** < 0.001**	5.91	5.66 to 6.16	** < 0.001**	5.67	5.41–5.93	** < 0.001**
Time^2^	1.1	0.75–1.45	** < 0.001**	0.69	0.38–0.99	** < 0.001**	0.54	0.28–0.80	** < 0.001**	0.53	0.26 to 0.79	** < 0.001**	0.59	0.34–0.84	** < 0.001**
Time^3^	0.61	0.24–0.97	**0.001**	0.45	0.13–0.76	**0.006**	0.37	0.10–0.64	**0.007**	0.18	− 0.10 to 0.45	0.208	0.31	0.05–0.57	**0.019**
Random effects															
σ^2^	0.95			0.73			0.52			0.55			0.48		
τ_00_	0.59_respondent_id_			0.77_respondent_id_			0.62_respondent_id_			0.44_respondent_id_			0.57_respondent_id_		
ICC	0.38			0.51			0.54			0.44			0.54		
N	63_respondent_id_			63_respondent_id_			63_respondent_id_			63_respondent_id_			63_respondent_id_		
Observations	177			177			177			177			177		
Marginal R^2^/conditional R^2^	0.120/0.455			0.053/0.540			0.044/0.563			0.048/0.471			0.054/0.568		

Predictors	Seek resources			Intervene			Report			Encourage others					
	Estimates	CI	p	Estimates	CI	p	Estimates	CI	p	Estimates	CI	p			
(b) Behavioural intention															
(Intercept)	4.35	3.90 to 4.79	** < 0.001**	5.49	5.08 to 5.90	** < 0.001**	5.55	5.15–5.95	** < 0.001**	5.49	5.10 to 5.88	** < 0.001**			
Time^2^	1.32	0.83 to 1.82	** < 0.001**	0.86	0.36 to 1.37	**0.001**	0.91	0.43–1.39	** < 0.001**	0.85	0.38 to 1.32	** < 0.001**			
Time^3^	0.47	− 0.04 to 0.99	0.071	0.43	− 0.09 to 0.95	0.103	0.58	0.09–1.07	**0.022**	0.37	− 0.11 to 0.86	0.126			
Random effects															
σ^2^	1.91			1.95			1.77			1.69					
τ_00_	1.15_respondent_id_			0.63_respondent_id_			0.75_respondent_id_			0.64 _respondent_id_					
ICC	0.38			0.25			0.3			0.27					
N	63_respondent_id_			63_respondent_id_			63_respondent_id_			63 _respondent_id_					
Observations	177			177			177			177					
Marginal R^2^/conditional R^2^	0.092/0.434			0.047/0.281			0.054/0.335			0.051 / 0.311					

p values in bold denote statistical significance.

### Does participation in the training increase participants’ actions to create inclusive, safe field environments?

*H2a. Post-training prevention behaviour (self-reported) and behavioural intention will increase significantly in the intervention group compared to the control.*

There were no differences detected in our linear regressions between treatment and control groups for any form of behavioural intention (Fig. 1b), nor for most forms of self-reported behaviour, except for intent to encourage others to take action, which was significantly greater for the treatment group versus control (β = 0.49, 95% CI [0.04, 0.94], p = 0.034, Table 2, Fig. 1c). However, scores for these questions were extremely low with little variation for both time points and treatment groups, suggesting floor effects (questions measured frequency of intent to act, with means in the range of 1.15–1.57 on a seven-point scale). Thus, we reject Hypothesis 2a.

*H2b. Changes in behavioural intention will not be sustained over time.*

Although we detected almost no differences between treatment and control groups, within-subjects comparisons revealed significant increases in all forms of behavioural intention immediately after the training (β = 0.85–1.32, 95% CI [range 0.36–1.82], p < 0.001, Table 3, Fig. 2b). Unlike results for knowledge and self-efficacy, significant increases in behavioural intention were sustained 1–2 months after the training delivery only for reporting intention (β = 0.58, 95% CI [0.09, 1.07], p = 0.02), while prevention, intervention, and encouragement intention all returned to not significantly different from baseline levels. These results indicate support for Hypothesis 2b for all forms of behavioural intention except reporting.

#### Does the training work equally well for all demographic groups?

*H3a. Increases in post-training knowledge, self-efficacy, and behavioural intention will be higher for women and gender minorities compared to men.*

**Figure 3:**
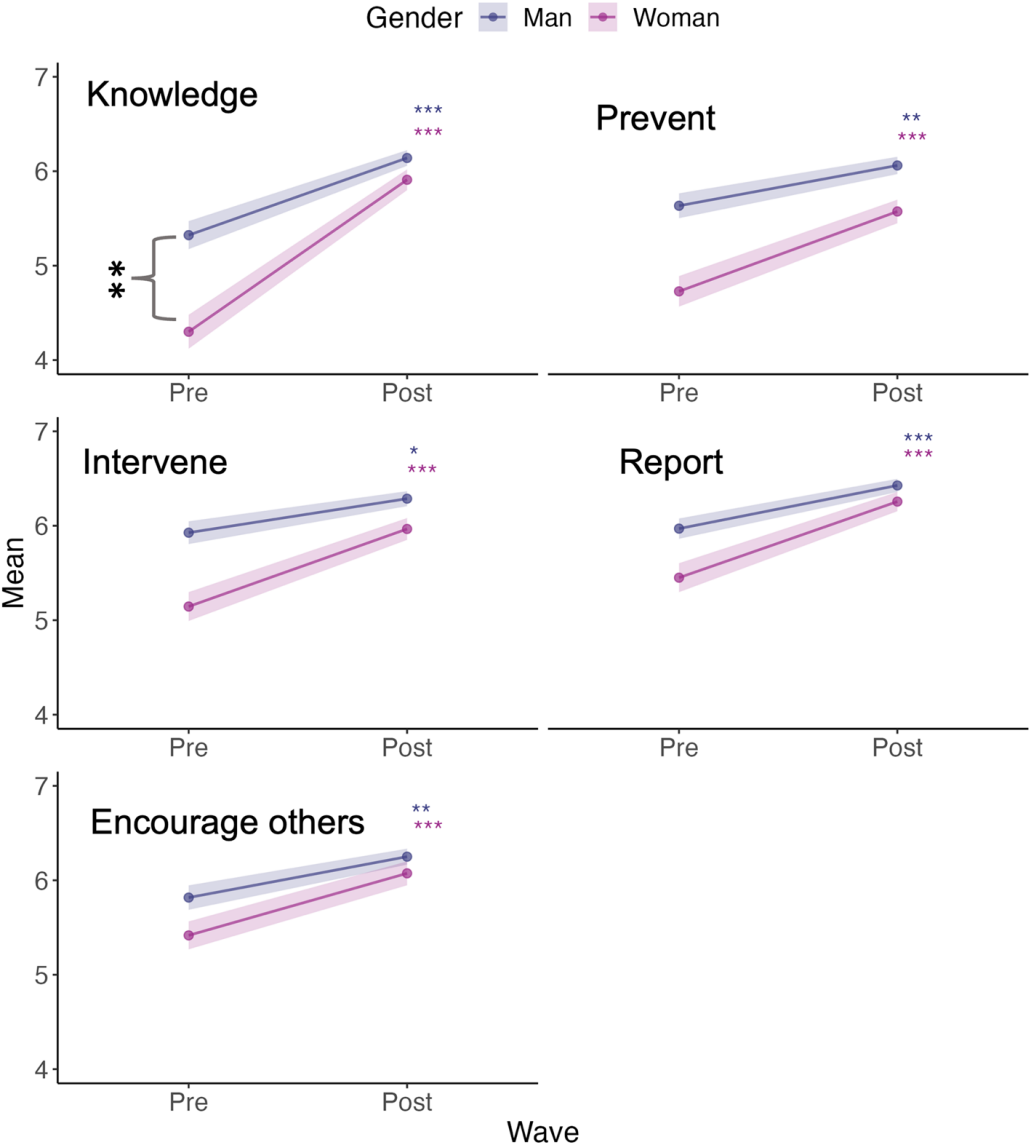
Change in scores related to knowledge and self-efficacy for men (n = 82) and women (n = 88) pre-training compared to post-training. Colored asterisks denote significant differences between scores within gender groups; black asterisks denote significant differences in changes in scores between gender groups (e.g. interaction effect).

Our within-subjects comparisons indicated that increases in knowledge immediately after the training were higher for women (n = 88) than men (n = 82) (β = 0.8, p = 0.004, Table 4, Fig. 3). However, there was no detectable gender difference in changes for scores related to self-efficacy and behavioural intention (Fig. 3a,b). Thus, Hypothesis 3a can be accepted for knowledge but not for self-efficacy or behavioural intention.

**Table 4:** Linear model results comparing pre-training (Time 1) and post-training (Time 2) responses among gender (n = 173) and race (n = 165) groups for survey questions for sexual harassment and assault related to knowledge, self-efficacy and behavioural intention.

Group	Concept	Variable	Term	β	CI lower	CI upper	Std. error	p value	Sig
Gender	Knowledge	Knowledge	Intercept:man	5.323	5.048	5.598	0.140	0.000	***
			Post-training	0.817	0.428	1.206	0.198	0.000	***
			Woman	− 1.023	− 1.404	− 0.643	0.193	0.000	***
			Post-training:woman	0.792	0.252	1.332	0.274	0.004	**
	Self- efficacy	Preventing	Intercept:man	5.634	5.371	5.898	0.134	0.000	***
			Post-training	0.427	0.054	0.800	0.189	0.025	**
			Woman	− 0.906	− 1.271	− 0.542	0.185	0.000	***
			Post-training:woman	0.419	− 0.097	0.936	0.263	0.111	
		Intervening	Intercept:man	5.927	5.683	6.171	0.124	0.000	***
			Post-training	0.360	0.015	0.704	0.175	0.041	*
			Woman	− 0.782	− 1.119	− 0.445	0.171	0.000	***
			Post-training:woman	0.462	− 0.016	0.940	0.243	0.058	
		Reporting	Intercept:man	5.970	5.740	6.199	0.117	0.000	***
			Post-training	0.457	0.133	0.782	0.165	0.006	**
			Woman	− 0.520	− 0.837	− 0.202	0.161	0.001	**
			Post-training:woman	0.348	− 0.101	0.798	0.229	0.128	
		Encouraging others	Intercept:man	5.817	5.565	6.069	0.128	0.000	***
			Post-training	0.433	0.077	0.789	0.181	0.017	*
			Woman	− 0.400	− 0.749	− 0.052	0.177	0.024	*
			Post-training:woman	0.224	− 0.270	0.718	0.251	0.372	
	Behavioural intention	Seeking resources	Intercept:man	4.293	3.882	4.703	0.209	0.000	***
			Post-training	0.720	0.139	1.300	0.295	0.015	*
			Woman	0.005	− 0.564	0.574	0.289	0.986	
			Post-training:woman	0.202	− 0.602	1.006	0.409	0.622	
		Intervening	Intercept:man	5.177	4.788	5.566	0.198	0.000	***
			Post-training	0.829	0.279	1.379	0.280	0.003	**
			Woman	0.222	− 0.317	0.761	0.274	0.418	
			Post-training:woman	− 0.155	− 0.917	0.607	0.388	0.689	
		Reporting	Intercept:man	5.287	4.899	5.674	0.197	0.000	***
			Post-training	0.817	0.269	1.365	0.279	0.004	**
			Woman	0.202	− 0.335	0.739	0.273	0.459	
			Post-training:woman	− 0.126	− 0.885	0.633	0.386	0.744	
		Encouraging others	Intercept:man	5.012	4.632	5.392	0.193	0.000	***
			Post-training	0.817	0.280	1.355	0.273	0.003	**
			Woman	0.252	− 0.275	0.779	0.268	0.348	
			Post-training:woman	− 0.182	− 0.927	0.563	0.379	0.631	
Race	Knowledge	Knowledge	Intercept:white	4.941	4.699	5.184	0.123	0.000	***
			Post-training	1.218	0.875	1.561	0.174	0.000	***
			URM	− 0.468	− 0.886	− 0.050	0.212	0.028	*
			Post-training:URM	0.118	− 0.475	0.712	0.302	0.695	
	Self-efficacy	Preventing	Intercept:white	5.279	5.045	5.514	0.119	0.000	***
			Post-training	0.630	0.298	0.962	0.169	0.000	***
			URM	− 0.404	− 0.809	0.000	0.206	0.050	*
			Post-training:URM	0.132	− 0.443	0.706	0.292	0.653	
		Intervening	Intercept:white	5.640	5.423	5.856	0.110	0.000	***
			Post-training	0.588	0.281	0.895	0.156	0.000	***
			URM	− 0.390	− 0.764	− 0.016	0.190	0.041	*
			Post-training:URM	0.090	− 0.441	0.620	0.270	0.740	
		Reporting	Intercept:white	5.887	5.692	6.083	0.100	0.000	***
			Post-training	0.585	0.308	0.863	0.141	0.000	***
			URM	− 0.557	− 0.895	− 0.219	0.172	0.001	**
			Post-training:URM	0.202	− 0.278	0.683	0.244	0.407	
		Encouraging others	Intercept:white	5.766	5.551	5.981	0.109	0.000	***
			Post-training	0.557	0.253	0.861	0.155	0.000	***
			URM	− 0.516	− 0.887	− 0.145	0.189	0.007	**
			Post-training:URM	0.120	− 0.406	0.647	0.268	0.653	
	Behavioural intention	Seeking resources	Intercept:white	4.090	3.742	4.438	0.177	0.000	***
			Post-training	0.978	0.485	1.472	0.251	0.000	***
			URM	0.713	0.112	1.315	0.306	0.020	*
			Post-training:URM	− 0.373	− 1.226	0.481	0.434	0.391	
		Intervening	Intercept:white	5.059	4.728	5.389	0.168	0.000	***
			Post-training	1.082	0.614	1.551	0.238	0.000	***
			URM	0.665	0.094	1.235	0.290	0.023	*
			Post-training:URM	− 0.815	− 1.625	− 0.004	0.412	0.049	*
		Reporting	Intercept:white	5.185	4.855	5.515	0.168	0.000	***
			Post-training	1.056	0.589	1.524	0.238	0.000	***
			URM	0.601	0.031	1.171	0.290	0.039	*
			Post-training:URM	− 0.787	− 1.596	0.021	0.411	0.056	
		Encouraging others	Intercept:white	5.000	4.678	5.322	0.164	0.000	***
			Post-training	1.005	0.548	1.461	0.232	0.000	***
			URM	0.491	− 0.065	1.047	0.283	0.083	
			Post-training:URM	− 0.723	− 1.512	0.067	0.401	0.073	

Asterisk denote statistically significant p value (*p < 0.05; **p < 0.01; ***p < 0.001).

No respondents reported identifying as non-binary, transgender, or other gender minorities; thus effects for other gender minorities could not be investigated.

*H3b. When controlling for gender, no significant differences will be observed in post-training outcomes based on age, race/ethnicity, role, region, or time at the agency.*

Within-subjects linear regressions comparing pre- and post-training data failed to detect significant differences in change in knowledge, self-efficacy, or behavioural intention based on age, race/ethnicity, education level, tenure at CDFW, occupation, or region when controlling for gender (p > 0.05, Table S4, n = 196, Fig. S2). Thus we find support for hypothesis H3b.

*H3c. Increases in post-training behavioural intention will be higher for staff who reported higher levels of pre-training prevention behaviour and prevention personal norms compared to their less engaged and committed peers.*

We used a correlated composite variable for self-reported pre-training prevention behaviour (Cronbach’s alpha = 0.91). Due to the prevalence of scores centered around the mean (score = 1.18), the data was categorized into two groups: “high prevention behaviour” (composite score > 1.2; n = 59) and “low prevention behaviour” (composite score < 1.2; n = 121). Using a correlated composite value for personal norms related to both harassment and assault (Cronbach’s alpha = 0.96), respondents were divided around the mean (composite score 6.13) into “high personal norm” (composite score range 6.25–7; n = 98) and “low personal norm” groups (composite score range 4–6.13; n = 78).

No differences in the change in behavioural intention were detected in our moderation analyses (linear regressions) between the high prevention behaviour and low prevention behaviour groups (β = − 0.26 to 0.26, 95% CI [range − 0.85 to 0.86], Table S5). Similarly, no differences were detected between norm groups for change in behavioural intention after the training (β = − 0.38 to 0.36, 95% CI [range − 0.9 to 0.6], Table S5). Thus, we can reject hypothesis H3c.

#### Do reporting rates increase after participants receive information about sexual harassment and assault?

*H4a. Post-training confidence in reporting and likelihood to report an incident of sexual harassment and assault will be higher in post-training surveys than pre-training.*

Reporting self-efficacy and behavioural intention were not significantly different between treatment and control groups (Fig. 1). However, our mixed effects models found that within subjects, both reporting self-efficacy (β = 0.53, 95% CI [0.26, 0.79], p < 0.001) and intention (β = 0.91, 95% CI [0.43, 1.39], p < 0.001) increased immediately after training (Table 3, Fig. 2). While this increase was sustained for reporting intention (β = 0.58, 95% CI [0.09, 1.07], p = 0.02), reporting self-efficacy returned to baseline 1–2 months after training (β = 0.18, 95% CI [− 0.10, 0.45] p = 0.208), Table 3, Fig. 2). These results partially support H4a.

On the other hand, sexual harassment and assault incident report data provided by CDFW indicated only one sexual harassment incident complaint was filed from April to August the year prior to the study period (2021). During the same period the following year (2022, the study period), three sexual harassment complaints were filed to CDFW, though only one of these complaints was filed by an employee who had participated in a BBFF training. The small number of incident reports prevented quantitative analyses of the change in reports filed.

### Exploratory analyses

Because of small sample sizes for RQ1, RQ2, and RQ4, we ran additional linear regressions using pre-post training data (n = 196) as a validity check for our results. Consistent with previous pilot data, results indicated significant increases in all forms of knowledge (β = 1.17, 95% CI [0.9, 1.42], p < 0.001), self-efficacy (β = 0.53–0.6, 95% CI [range 0.29–0.85], p < 0.001), and behavioural intention (β = 0.72–0.87, 95% CI [range 0.38–1.2], p < 0.001) immediately after the training compared to participants’ pre-training scores (Table S6).

#### Individual variability in the effect of the training

Within-subjects longitudinal comparisons suggested considerable individual variability in predicted effects of the training on knowledge, self-efficacy, and behavioural intention that were not captured by the fixed effects of the models (random effects ranging from τ00 = 0.44–1.15; Table 3). To further investigate this variability, we conducted more detailed analyses of gender and race particularly for changes in knowledge, self-efficacy, and behavioural intention.

#### Effects of race and gender

In addition to gender differences in score change (RQ3), we investigated the effect of gender on within-subjects responses for knowledge and self-efficacy. While both men (n = 82) and women (n = 91) demonstrated significant increases post-training in all forms of knowledge and self-efficacy compared to pre-training (β = 0.4–0.82, 95% CI [range 0.02–1.21], p < 0.05, Table 4), both before and after training, women consistently reported significantly lower scores than men for both knowledge (β = -1.02, 95% CI [− 1.4, − 0.64], p < 0.001) and self-efficacy (β = − 0.43 and − 0.91, 95% CI [range − 1.27 to − 0.05], p < 0.05, Table 4, Fig. 3). There was no significant difference between genders for scores related to behavioural intention.

**Figure 4:**
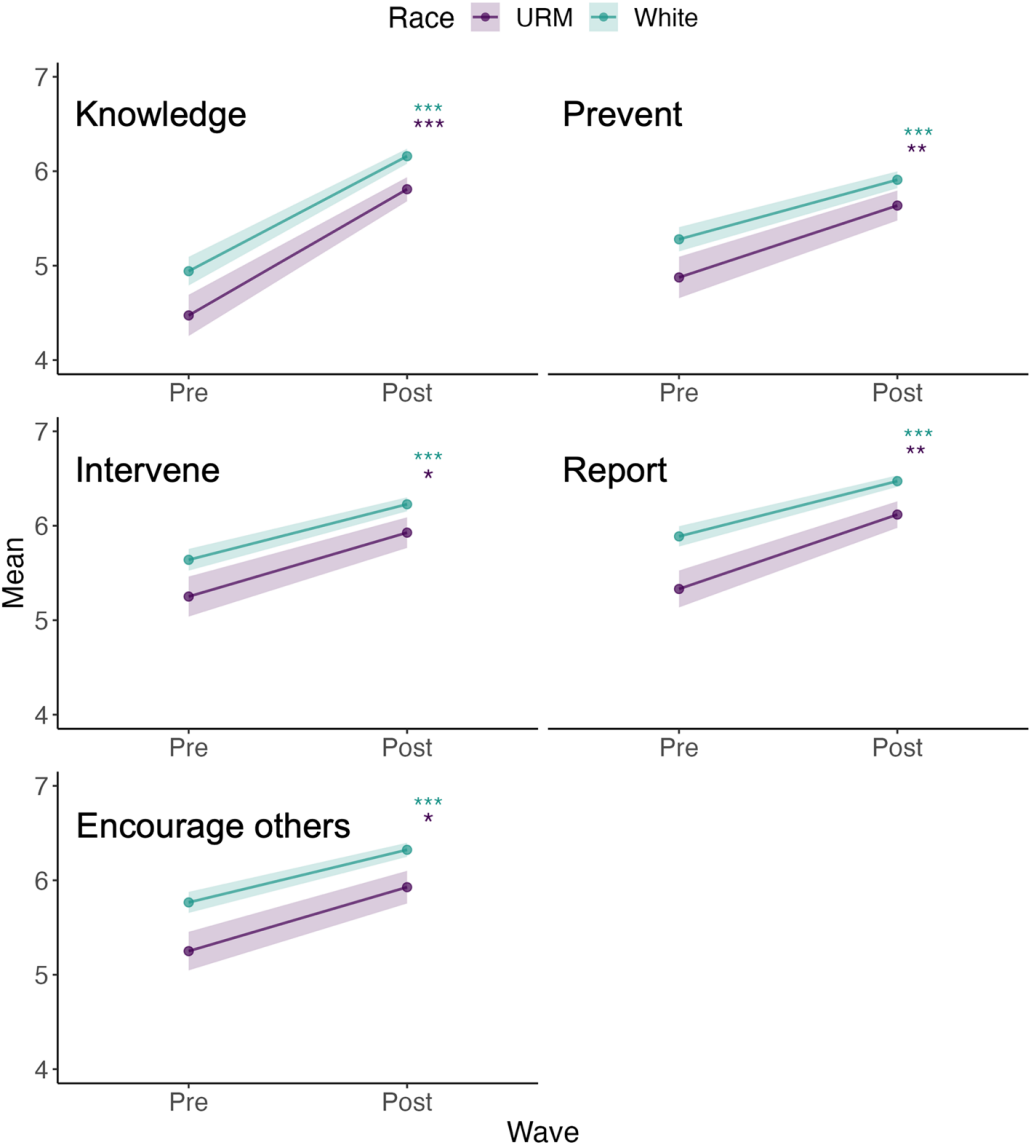
Change in within-subjects scores related to knowledge and self-efficacy for underrepresented minority (URM, n = 55) and white respondents (n = 110) pre-training compared to post-training. Colored asterisks denote significant changes between pre-training and post-training scores within each gender group.

We also investigated the effect of race and ethnicity on within-subjects pre- and post-training responses for knowledge, self-efficacy, and behavioural intention. We compared respondents who identified as white (n = 110) to those with one or more non-white racial or ethnic identities, grouped together as underrepresented minority (URM) respondents (n = 55). While both groups reported significant increases in knowledge and self-efficacy from pre-training to post-training (β = 0.59–1.22, 95% CI [range 0.25–1.56, p < 0.001), URM respondents consistently reported significantly lower scores than white respondents for knowledge (β = − 0.47, 95% CI [− 0.89, − 0.05], p = 0.028) and all forms of self-efficacy (β = − 0.4 to − 0.6, 95% CI [range − 0.90, 0], p < 0.05, Table 4, Fig. 4). On the other hand, URM respondents reported higher behavioural intention than white respondents for intervening (β = 0.67, 95% CI [0.09, 1.24], p = 0.023) and reporting (β = 0.60, 95% CI [0.03, 1.17], p < 0.05, Table 4).

## Discussion

### Training appears to boost sexual harassment and assault prevention knowledge and self-efficacy, but effects on behaviour remain unclear

Sexual and gender-based harassment and assault are pervasive in scientific and natural resource fieldwork, but the recent development of training and intervention programs seeks to reduce its prevalence and empower field scientists and students. However, many training programs seeking to reduce harassment in the workplace fail to produce results, and many have even backfired because they induce defensiveness, greater acceptance of harassing behaviours in perpetrators, and/or retaliation against victims who complain, leading to worker disaffection and turnover^33,45^. In this study, we examined the impact of an interactive, peer-based, fieldwork-focused harassment and assault prevention training program delivered to staff of CDFW, a US state natural resource agency. Our findings demonstrate both immediate increases and longer-term persistence in three established precursors of action: self-reported knowledge, self-efficacy, and to a lesser extent behavioural intention. These results suggest the potential positive impact of a relatively short-duration training program in contributing to broader organizational efforts to end harassment and assault risk within the high-risk setting of scientific fieldwork.

While these results suggest promising outcomes for post-training increases in knowledge- and self-efficacy, the long-term effect of the training on behavioural intention was weaker, a pattern that is aligned with other assessments of training interventions^46^. This aligns with other research that has identified that knowledge and self-efficacy are necessary but not sufficient precursors to behavioural intention, so training efforts may need to consider other constructs such as social norms, attitudes and identity to impact behavioural intention^41,47^. Given that the BBFF training we assessed lasts only 90 min, further research is needed to investigate the effects of longer-duration trainings; in particular, multiple studies have suggested four hours as a minimum duration for long-term training effectiveness^48–50^ (though other studies have suggested that diversity training content is more important than duration in determining outcomes^33^). Regardless, these short-duration trainings can be viewed as a way to initiate deeper efforts towards harassment and assault prevention action, as they help build knowledge and self-efficacy among participants toward an immediate goal of behaviour change and an ultimate goal of organizational culture change. Our results further suggest that trainings that seek to improve bystander efficacy may be strengthened by integrating mastery experiences, knowledge-based interventions, and social modeling^36^.

### The role of race, gender, and past experience in training outcomes

We did not detect differences in training outcomes based on gender, race, or other individual-level characteristics. However, we did find that women score lower than men on most within-subjects metrics, and URM individuals scored lower than white participants for knowledge and self-efficacy. In other words, although men and women benefitted similarly from the training, women’s scores started lower and remained lower after training This pattern could be explained by an underestimation of the difficulty of taking action in response to incidents of harassment and assault by individuals who have little or no experience with the issue (i.e. the Dunning–Kruger effect)^51^. While women, particularly women of color, are most likely to experience and report sexual harassment^52,53^, men have been shown to be more likely to characterize harassment incorrectly^54^ and less quickly^55^, less likely to believe harassment complaints, and more likely to respond poorly to harassment prevention training programs^56^. Just like other social problems related to stigma and discrimination, people who are not directly impacted may be less likely to experience or see the problem, and therefore less likely to grasp the difficulty of responding to it. In addition, the lower reported scores for women and URM individuals may be connected to greater levels of mistrust in their institutions, for example if they have previously experienced or witnessed a negative outcome as a result of the reporting process^57^.

These gender and race patterns can be viewed within the broader literature that connects sexual or gender-based harassment to larger and intersectional issues of power, wherein identity-based harassment is used as an expression of dominance and a tool to enforce or protect an individual’s privileged sex-, race- or other identity-based social status within socially stratified and inequal systems^6,50,58–60^. These results point to a broader framing of harassment training efforts not only as a method to prevent incidents, but also as one tool in a more expansive effort to dismantle systemic power imbalances and pursue equity and justice in science and academia.

Surprisingly, personal norms and past behaviour did not mediate the impact of the training on behavioural intention, suggesting that the training is not only impactful for people already interested in or motivated by the topic. This is promising, given our demographic results showing lower scores reported for marginalized groups, and suggests that this kind of training can be helpful to people at different stages of knowledge about and experience with harassment.

However, our sample sizes, particularly for the number of URM respondents (n = 55) compared to 110 white respondents in our within-subjects sample, limits the generalizability of these results, and may be reflective of broad underrepresentation of marginalized racial and ethnic groups in governmental natural resources management as a whole^61–63^. The fact that no survey respondents identified as non-binary, transgender, or any other gender identity besides man or woman limits our understanding of how these trainings affect gender minorities, especially given recent evidence about the heightened risk of harassment for this population in the field and in general^64–66^. We also did not collect data on sexual orientation or disability^66,67^, gaps that could be filled by follow-up studies. Still, these findings indicate that to the extent that the training worked, it worked equally for these race and gender groups, and that the patterns observed for race and gender exist within participants regardless of training. Future training efforts could consider developing customized trainings that meet the needs and existing knowledge of different groups. It is also possible that women and URM respondents are underreporting their capabilities, and men and white respondents are over-reporting their capabilities. In situations where this is the case, future trainings could integrate our results within training content to support more accurate self-appraisals.

### Implications for organizational initiatives

This study suggests that large state agencies (particularly those with high need for fieldwork activities) are useful platforms for deploying and testing harassment prevention programs, given their access to large numbers of field-going staff. There is building evidence that the natural resource fields present obstacles to people with marginalized identities, and agencies will continue to face greater pressure to implement solutions^68–70^. This kind of interactive, peer-led training program could be replicated, tailored, and improved at other large state and federal agencies.

Our results also suggest limitations related to incident reporting that can be addressed by institutions. While we found that the training had positive and sustained immediate effects on reporting self-efficacy and intention, only three reports related to sexual harassment or assault were made to CDFW during the study period. This prevented rigorous analysis of actual reporting rates. Further, the low number of reports points to the inherent difficulty in using reporting rates as indicators of actual incident rates and responses^45,56,71^. The low number of reports is likely associated with the harmful effects of the primary reporting systems used by most large academic and research institutions (including CDFW)—in particular, universal mandatory reporting, which requires employees to report any incident of sexual harassment or misconduct they learn about to officials, even against a victim’s consent^72^. Mandatory reporting without consent has been demonstrated to discourage survivors from disclosing incidents and conflict with survivors’ healing processes^73^, and it is unlikely to result in justice in the form of sanctions for the perpetrator^74^. Even worse, mandatory reporting can lead to retaliatory behaviour from alleged perpetrators^75,76^ and other coworkers in as many as 63% of workplace sexual harassment cases^76^. Alternative reporting systems to mandatory reporting have been suggested, including a shift toward “mandatory supporting” that prioritize confidential reporting options, require consent for official reports, and provide trauma-informed training so that employees can support survivors who do disclose^72^. These changes will be particularly important in the high-risk setting of fieldwork and at government agencies where reporting rates, as this study demonstrates, can be extremely low.

Efforts to improve the organizational climate of fieldwork settings, like that tested by this study, offer an alternative to reporting-focused initiatives with the goal of legal compliance rather than the elimination of harassment. However, it is important to note than real workplace climate improvement is not achievable by individual participation in trainings alone, and requires substantial and sustained institutional commitment. Agencies and other large institutions could fulfill their responsibilities towards staff safety by developing and funding efforts that reprimand and remove offenders and build effective, trauma-informed reporting systems that actually support and protect victims. Without institutional commitment, training programs that only target employees and do not tackle larger institutional barriers to inclusion risk sending mixed messages and can foist the burden of systemic change on individuals with the least power.

### Study limitations and future research

In addition to sample size and demographic limitations described above, working within a large governmental agency provided both challenges and opportunities for future research and recommendations. Obtaining control data (participants who completed surveys without being trained) was challenging due to the difficulty of incentivizing Time 1 survey completion for participants who were not scheduled to take the training until months later. One additional possibility is that the receipt of the Time 1 survey prompted individuals to sign up for the training itself, introducing potential biases in the order that participants completed trainings. As a result, our final sample sizes were 50% of desired sizes for the treatment group and 10% for the control group, which limited the power of our treatment–control analyses to detect small and moderate effects. We also struggled to retain participants for longitudinal analyses (n = 64 out of 925 staff trained). Future research should strive to incorporate rigorous control groups and improve survey recruitment processes to allow for deeper demographic analyses. One way to achieve this might be to use a control intervention, like a “traditional” online training, that prompts participants to take the first survey wave, which could increase the control group survey sample. Also, providing incentives for survey completion like small prizes could help increase completion rates.

Our attempt to measure self-reported behaviour through a question about frequency (with options from “once a day or more” to “never”, Table S1) likely led to floor effects, with nearly all respondents choosing the lowest possible option, i.e. that they never did the behaviour while working in the field. This made it challenging to obtain robust responses related to self-reported behaviours. This challenge is inherent to sexual harassment and assault prevention research, as bystanders might only be called to take key actions once a field season—for instance, in the creation of a field safety plan. Future survey instruments could focus on more frequent “lower-level” behaviours that might be precursors to more direct forms of sexual harassment and assault prevention, such as actions to create inclusive organizational fieldwork climates. For example, survey questions that ask whether participants engaged in a community agreement exercise, or intervened to address microaggressions or expressions of implicit bias could be additional indicators of organizational climate. Recognizing the limitations to measuring behaviours through surveys, a second approach might be to integrate data collection about prevention behaviours into pre-existing organizational systems, such as by refining performance evaluations to measure and reward behaviours that promote inclusive cultures.

Interventions that help participants identify and diagnose a spectrum of exclusionary behaviours in others (e.g., self-protectionism, or defensive behaviour to protect one's perceived advantages) can also help elucidate how to take action toward building more holistic, inclusionary behaviours that promote belonging and psychological safety in fieldwork^77^.

## Conclusion

This study provides support for the utility of an interactive, scenario-based training intervention for field-based staff and scientists at a large state agency, and can be a model for other large institutions looking to move beyond click-through online modules toward more interactive modes of harassment prevention training. Training should not be thought of as a panacea, but rather, especially in light of our results, as a way to open the door for larger conversations about organizational climate and inclusive settings, especially in the high-risk setting of scientific fieldwork.

### Supplementary Information

Supplementary Information.

## Data Availability

De-identified data is publicly available at: https://github.com/mcucsc/harassment_survey_cdfw. This data does not include demographic information for respondents. Please contact the corresponding author for demographic data, which is available upon reasonable request.

## Code availability

Code use in this study is available at https://github.com/mcucsc/harassment_survey_cdfw.

## References

[1] Cotton DRE (2009). Field biology experiences of undergraduate students: The impact of novelty space. J. Biol. Educ..

[2] Allen-Ramdial S-AA, Campbell AG (2014). Reimagining the pipeline: Advancing STEM diversity, persistence, and success. Bioscience.

[3] Beltran RS (2020). Field courses narrow demographic achievement gaps in ecology and evolutionary biology. Ecol. Evol..

[4] Clancy KBH, Nelson RG, Rutherford JN, Hinde K (2014). Survey of academic field experiences (SAFE): Trainees report harassment and assault. PLoS One.

[5] McLaughlin H, Uggen C, Blackstone A (2012). Sexual harassment, workplace authority, and the paradox of power. Am. Sociol. Rev..

[6] Täuber S, Mahmoudi M (2022). How bullying becomes a career tool. Nat. Hum. Behav..

[7] Posselt JR (2020). Equity in Science: Representation, Culture, and the Dynamics of Change in Graduate Education.

[8] Nordling L (2019). Codes of conduct aim to curb harassment at field sites. Science.

[9] Diep, F. ‘I Was Fed Up’: How #BlackInTheIvory Got Started, and What Its Founders Want to See Next. *The Chronicle of Higher Education* https://www.chronicle.com/article/i-was-fed-up-how-blackintheivory-got-started-and-what-its-founders-want-to-see-next (2020).

[10] *Sexual Harassment of Women: Climate, Culture, and Consequences in Academic Sciences, Engineering, and Medicine*. https://www.nap.edu/catalog/24994 (2018). 10.17226/24994. 29894119

[11] Figley C (2012). Encyclopedia of Trauma: An Interdisciplinary Guide.

[12] Fitzgerald LF, Cortina LM, Travis CB (2018). Sexual harassment in work organizations: A view from the 21st century. APA handbook of the Psychology of Women: Perspectives on Women’s Private and Public Lives.

[13] Leskinen EA, Cortina LM (2014). Dimensions of disrespect: Mapping and measuring gender harassment in organizations. Psychol. Women Q..

[14] Karami A, Swan S, White CN, Ford K (2019). Hidden in plain sight for too long: Using text mining techniques to shine a light on workplace sexism and sexual harassment. Psychol. Violence.

[15] Berdahl J, Bhattacharyya B (2021). Four ways forward in studying sex-based harassment. Equal. Divers. Inclusion Int. J..

[16] Lindquist C, McKay T (2010). Sexual Harassment Experiences and Consequences for Women Faculty in Science, Engineering, and Medicine. In In RTI Press Policy Brief.

[17] Berdahl JL, Cooper M, Glick P, Livingston RW, Williams JC (2018). Work as a masculinity contest. J. Soc. Issues.

[18] Glick P, Berdahl JL, Alonso NM (2018). Development and validation of the masculinity contest culture scale: Development and validation of the masculinity contest culture scale. J. Soc. Issues.

[19] Jahnke SA (2019). The prevalence and health impacts of frequent work discrimination and harassment among women firefighters in the US fire service. Biomed. Res. Int..

[20] Huseth-Zosel A, Larson M, Nelson K (2021). Health effects of the #metoo movement by gender: Public health implications of a social movement. Women’s Stud. Int. Forum.

[21] Golding JM (1999). Sexual-assault history and long-term physical health problems: Evidence from clinical and population epidemiology. Curr. Dir. Psychol. Sci..

[22] Waigandt A, Wallace DL, Phelps L, Miller DA (1990). The impact of sexual assault on physical health status. J. Traum. Stress.

[23] Campbell R, Wasco S (2005). Understanding rape and sexual assault 20 years of progress and future directions. J. Interpersonal Violence.

[24] Bell SC, Coker AL, Clear ER (2019). Bystander program effectiveness: A review of the evidence in educational settings (2007–2018). Handb. Sex. Assault Sex. Assault Prev..

[25] Nash M (2019). “Antarctica just has this hero factor…”: Gendered barriers to Australian Antarctic research and remote fieldwork. PLoS One.

[26] McMahon S, Lowe Hoffman M, McMahon SM, Zucker S, Koenick RA (2013). What would you do? Strategies for bystander intervention to prevent sexual violence by college students. J. Coll. Char..

[27] Banyard VL (2008). Measurement and correlates of prosocial bystander behaviour: The case of interpersonal violence. Violence Vict..

[28] Banyard V, Moynihan M (2011). Variation in bystander behaviour related to sexual and intimate partner violence prevention: Correlates in a sample of college students. Psychol. Violence.

[29] Banyard VL, Plante EG, Moynihan MM (2004). Bystander education: Bringing a broader community perspective to sexual violence prevention. J. Community Psychol..

[30] Banyard VL (2011). Who will help prevent sexual violence: Creating an ecological model of bystander intervention. Psychol. Violence.

[31] Bandura, A. *Self-efficacy: Toward a Unifying Theory of Behavioural Change*. 10.1037//0033-295x.84.2.191 847061

[32] Bandura A (2000). Exercise of human agency through collective efficacy. Curr. Dir. Psychol. Sci..

[33] Roehling MV, Huang J (2018). Sexual harassment training effectiveness: An interdisciplinary review and call for research. J. Organ. Behav..

[34] Perry E, Kulik C, Field M (2009). Sexual harassment training: Recommendations to address gaps between the practitioner and research literatures. Human Resour. Manage..

[35] Bandura A, Schunk DH (1981). Cultivating competence, self-efficacy, and intrinsic interest through proximal self-motivation. J. Personal. Soc. Psychol..

[36] Geiger N, Swim J, Fraser J (2017). Creating a climate for change: Interventions, efficacy and public discussion about climate change. J. Environ. Psychol..

[37] Niemiec R, Jones MS, Lischka S, Champine V (2021). Efficacy-based and normative interventions for facilitating the diffusion of conservation behaviour through social networks. Conserv. Biol..

[38] Mujal GN, Taylor ME, Fry JL, Gochez-Kerr TH, Weaver NL (2021). A systematic review of bystander interventions for the prevention of sexual violence. Trauma Violence Abuse.

[39] Banyard V, Moynihan M, Cares A, Warner R (2014). How do we know if it works? Measuring outcomes in bystander-focused abuse prevention on campuses. Psychol. Violence.

[40] Morrison, S., Hardison, J., Mathew, A. & O’Neil, J. An Evidence-Based Review of Sexual Assault Preventive Intervention Programs: (513692006-001) (2004). 10.1037/e513692006-001.

[41] McMahon S (2015). Predicting bystander behaviour to prevent sexual assault on college campuses: The role of self-efficacy and intent. Am. J. Community Psychol..

[42] Orsini MM (2019). Bystander intervention training that goes beyond sexual violence prevention. Am. J. Health Stud..

[43] Sjögren B, Thornberg R, Wänström L, Gini G (2020). Associations between individual and collective efficacy beliefs and students’ bystander behaviour. Psychol. Sch..

[44] Bates D, Mächler M, Bolker B, Walker S (2015). Fitting linear mixed-effects models using lme4. J. Stat. Softw..

[45] Dobbin, F. & Kalev, A. *Why Sexual Harassment Programs Backfire* (2022).

[46] Roehling MV, Wu D, Choi MG, Dulebohn JH (2022). The effects of sexual harassment training on proximal and transfer training outcomes: A meta-analytic investigation. Person. Psychol..

[47] de Vries H, Dijkstra M, Kuhlman P (1988). Self-efficacy: The third factor besides attitude and subjective norm as a predictor of behavioural intentions. Health Educ. Res..

[48] Kalinoski Z (2013). A meta-analytic evaluation of diversity training outcomes. J. Organ. Behav..

[49] Nagy MS, Curl-Nagy DJ (2019). Workplace civility training: An antidote to traditional sexual harassment training. Ind. Organ. Psychol..

[50] Cortina LM, Areguin MA (2021). Putting people down and pushing them out: Sexual harassment in the workplace. Annu. Rev. Organ. Psychol. Organ. Behav..

[51] Dunning D, Olson JM, Zanna MP (2011). Chapter five—The Dunning–Kruger effect: On being ignorant of one’s own ignorance. Advances in Experimental Social Psychology.

[52] Clancy KBH, Lee KMN, Rodgers EM, Richey C (2017). Double jeopardy in astronomy and planetary science: Women of color face greater risks of gendered and racial harassment. J. Geophys. Res. Planets.

[53] Cassino D, Besen-Cassino Y (2019). Race, threat and workplace sexual harassment: The dynamics of harassment in the United States, 1997–2016. Gender Work Organ..

[54] Rothgerber H, Kaufling K, Incorvati C, Andrew CB, Farmer A (2021). Is a reasonable woman different from a reasonable person? Gender differences in perceived sexual harassment. Sex Roles.

[55] Rotundo M, Nguyen D-H, Sackett PR (2001). A meta-analytic review of gender differences in perceptions of sexual harassment. J. Appl. Psychol..

[56] Dobbin F, Kalev A (2019). The promise and peril of sexual harassment programs. Proc. Natl. Acad. Sci..

[57] Smith CP, Freyd JJ (2014). Institutional betrayal. Am. Psychol..

[58] Berdahl JL (2007). Harassment based on sex: Protecting social status in the context of gender hierarchy. AMR.

[59] Bondestam F, Lundqvist M (2020). Sexual harassment in higher education—a systematic review. Eur. J. Higher Educ..

[60] Täuber S, Loyens K, Oertelt-Prigione S, Kubbe I (2022). Harassment as a consequence and cause of inequality in academia: A narrative review. ClinicalMedicine.

[61] Haynes NA, Jacobson S (2015). Barriers and perceptions of natural resource careers by minority students. J. Environ. Educ..

[62] *Department of the Interior Fiscal Year 2018 Management Directive 715 Report*. https://www.doi.gov/sites/doi.gov/files/uploads/final-fy-2018-doi-md-715-signed_1.pdf (2018).

[63] CFI Group. *Department of the Interior Work Environment Survey January-March 2017*. https://www.doi.gov/sites/doi.gov/files/uploads/doi_wes_technical_report.pdf (2017).

[64] Bell, B., Avila, T., McLellan, O., Griffith, E. & Sawyer, A. Experiences of the LGBTQIA+ Community in the Earth and Planetary Sciences. **2021**, SY55B-0350 (2021).

[65] Kamran M, Jennings K (2023). Fieldwork and LGBTQ+ identities: Queering the outdoors. Integr. Comp. Biol..

[66] Wilkins K (2023). Sexual harassment disproportionately affects ecology and evolution graduate students with multiple marginalized identities in the United States. BioScience.

[67] Demery A-JC, Pipkin MA (2020). Safe fieldwork strategies for at-risk individuals, their supervisors and institutions. Nat. Ecol. Evol..

[68] Jones MS, Solomon J (2019). Challenges and supports for women conservation leaders. Conserv. Sci. Pract..

[69] Anderson, W. S. & Services, U.-A.-W. The Changing Face of the Wildlife Profession: Tools for Creating Women Leaders.

[70] Baruah B, Biskupski-Mujanovic S (2021). Navigating sticky floors and glass ceilings: Barriers and opportunities for women’s employment in natural resources industries in Canada. Nat. Resour. Forum.

[71] Ilies R, Scott BA, Judge TA (2006). The interactive effects of personal traits and experienced states on intraindividual patterns of citizenship behaviour. AMJ.

[72] Holland KJ, Hutchison EQ, Ahrens CE, Torres MG (2021). Reporting is not supporting: Why mandatory supporting, not mandatory reporting, must guide university sexual misconduct policies. Proc. Natl. Acad. Sci..

[73] Holland KJ, Cipriano AE, Huit TZ (2021). “A victim/survivor needs agency”: Sexual assault survivors’ perceptions of university mandatory reporting policies. Anal. Soc. Issues Public Policy.

[74] Richards TN, Gillespie LK, Claxton T (2021). Examining incidents of sexual misconduct reported to title IX coordinators: Results from New York’s Institutions of Higher Education. J. Sch. Viol..

[75] Harsey SJ, Freyd JJ (2022). Defamation and DARVO. J. Trauma Dissoc..

[76] Dahl, G. B. & Knepper, M. M. *Why is Workplace Sexual Harassment Underreported? The Value of Outside Options Amid the Threat of Retaliation*. http://www.nber.org/papers/w29248 (2021).

[77] Rodrigues MA, Mendenhall R, Clancy KBH (2021). ‘There’s realizing, and then there’s realizing’: How social support can counter gaslighting of women of color scientists. J. Women Minor. Sci. Eng..

