# Supplementary material for: Testing the effectiveness of interactive training on sexual harassment and assault in field science
Source: Sci Rep. 2024 Jan 8;14:523. doi: 10.1038/s41598-023-49203-0 (PMC10774269; doi:10.1038/s41598-023-49203-0)
Supplement: Supplementary file 1 — Supplementary Information. [file 41598_2023_49203_MOESM1_ESM.docx]

**Supplementary Information**

*Testing the effectiveness of interactive training on sexual harassment and assault in field science*

**Figures**


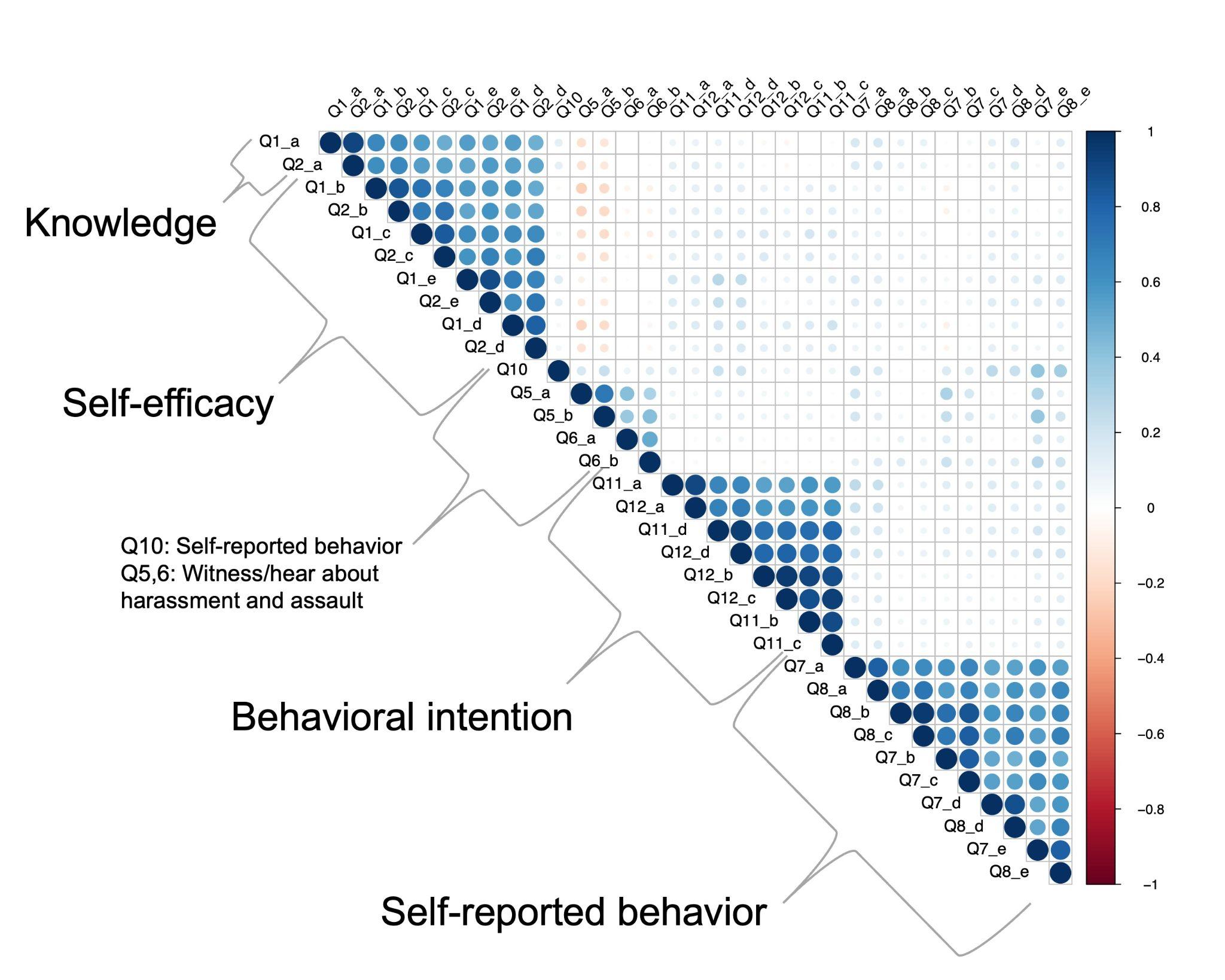


**Fig S1.** Correlation plot for responses for all variables, including questions asked separately for sexual harassment and sexual assault. Cronbach’s alpha greater than 0.8 was used to group variables asking the same question separately for harassment and assault. Question text details in Table S1.


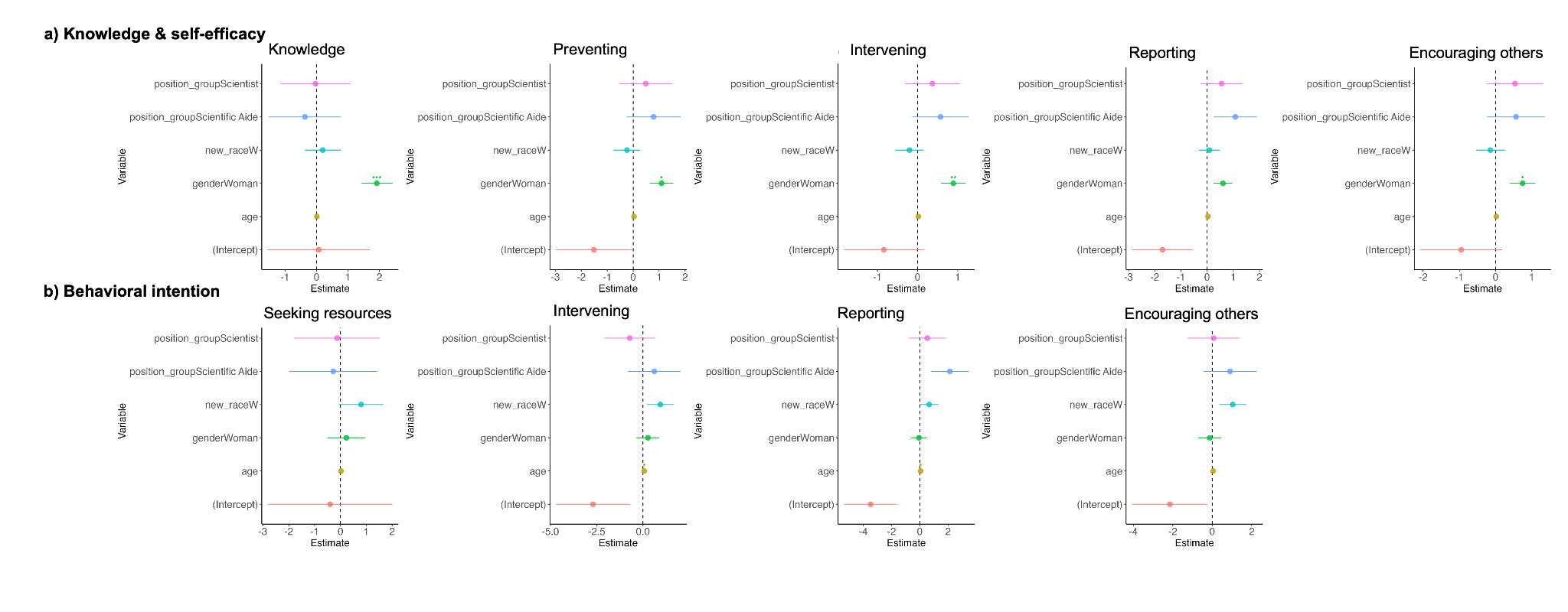


**Fig S2.** Effect sizes of predictor variables of model for within-subjects comparison of change in scores between within-subjects pre-training scores and post-training scores. Horizontal bars represent 95% confidence intervals around those estimates. P-values for each predictor are displayed on the plot, with asterisks indicating the level of statistical significance (*** for p < .001, ** for p < .01, and * for p < .05). Tenure (time at CDFW) and educational background are excluded from these models as they were both highly correlated with position at CDFW.

**Tables**

**Table S1.** Variables to be measured across three survey waves. All belief and behavioral statements measured on 7-point Likert scales unless noted otherwise, “sexual harassment/sexual assault” questions asked twice, once each for harassment or assault.

| **Wave asked** | **Question bucket** | **Question ID** | **Question text** | **Hypothesis** | **Construct** | **Response option** |
| --- | --- | --- | --- | --- | --- | --- |
| 1,2,3 | Q1. Please rate the degree to which you agree or disagree with each of the following statements regarding **sexual harassment** within your work at CDFW. | Q1_a | I feel knowledgeable about existing resources to help me prevent, intervene in, and report sexual harassment in field settings | H1a,  H1b | Knowledge | (7-point)  Strongly agree (7)  Agree (6)  Somewhat agree (5)  Neither agree nor disagree (4)  Somewhat disagree (3)  Disagree (2)  Strongly disagree (1) |
|  |  | Q1_b | I feel confident in my ability to prevent sexual harassment in field settings | H1a,  H1b | Self-efficacy |  |
|  |  | Q1_c | I feel confident in my ability to intervene in an incident of sexual harassment in a field setting | H1a,  H1b | Self-efficacy |  |
|  |  | Q1_d | I feel confident in my ability to report an incident of sexual harassment in a field setting | H1a,  H1b | Self-efficacy |  |
|  |  | Q1_e | I feel confident in my ability to encourage others to take action to prevent sexual harassment in field settings | H1a,  H1b | Self-efficacy |  |
| 1,2,3 | Q2. Please rate the degree to which you agree or disagree with each of the following statements regarding sexual assault within your work at CDFW. | Q2_a | I feel knowledgeable about existing resources to help me prevent, intervene in, and report sexual assault in field settings | H1a,  H1b | Knowledge | (7-point)  Strongly agree (7)  Agree (6)  Somewhat agree (5)  Neither agree nor disagree (4)  Somewhat disagree (3)  Disagree (2)  Strongly disagree (1) |
|  |  | Q2_b | I feel confident in my ability to prevent sexual assault in field settings | H1a,  H1b | Self-efficacy |  |
|  |  | Q2_c | I feel confident in my ability to intervene in an incident of sexual assault in a field setting | H1a,  H1b | Self-efficacy |  |
|  |  | Q2_d | I feel confident in my ability to report an incident of sexual assault in a field setting | H1a,  H1b | Self-efficacy |  |
|  |  | Q2_e | I feel confident in my ability to encourage others to take action to prevent sexual assault in field settings | H1a,  H1b | Self-efficacy |  |
| 1,2 | Q3. Please rate the degree to which you agree or disagree with each of the following statements about **sexual harassment** prevention at CDFW. | Q3_a | I personally feel a moral obligation to take action to prevent sexual harassment in field settings at CDFW | H1a | Personal norm | (7-point)  Strongly agree (7)  Agree (6)  Somewhat agree (5)  Neither agree nor disagree (4)  Somewhat disagree (3)  Disagree (2)  Strongly disagree (1) |
|  |  | Q3_b | I am confident that together we, as CDFW, can prevent sexual harassment in field settings | H1a | Collective efficacy |  |
|  |  | Q3_c | I am sure that we, as CDFW, can achieve progress towards preventing sexual harassment in field settings, because we are all pulling in the same direction | H1a | Collective efficacy |  |
| 1,2 | Q4. Please rate the degree to which you agree or disagree with each of the following statements about sexual assault prevention at CDFW. | Q4_a | I personally feel a moral obligation to take action to prevent sexual assault in field settings at CDFW |  | Personal norm | (7-point)  Strongly agree (7)  Agree (6)  Somewhat agree (5)  Neither agree nor disagree (4)  Somewhat disagree (3)  Disagree (2)  Strongly disagree (1) |
|  |  | Q4_b | I am confident that together we, as CDFW, can prevent sexual assault in field settings |  | Collective efficacy |  |
|  |  | Q4_c | I am sure that we, as CDFW, can achieve progress towards preventing sexual assault in field settings, because we are all pulling in the same direction |  | Collective efficacy |  |
| 1,3 | Q5 While you are working in the field for CDFW, approximately how often do you: | Q5_a | Witness behavior you consider sexual harassment |  | Observed behavior | (7-point)  Once a day or more (7)  Once a week (6)  Once a month (5)  Once every 2-3 months (4)  Once every six months (3)  Once a year (2)  Never (1) |
|  |  | Q5_b | Hear about behavior you consider sexual harassment |  |  |  |
| 1,3 | Q6. While you are working in the field for CDFW, approximately how often do you: | Q6_a | Witness behavior you consider sexual assault | H2a | Observed behavior | (7-point)  Once a day or more (7)  Once a week (6)  Once a month (5)  Once every 2-3 months (4)  Once every six months (3)  Once a year (2)  Never (1) |
|  |  | Q6_b | Hear about behavior you consider sexual assault | H2a |  |  |
| 1,3 | Q7 While you are working in the field for CDFW, approximately how frequently do you do the follow actions in response to **sexual harassment**? | Q7_a | Seek out resources about how to prevent, intervene in, or report sexual harassment in a field setting | H2a | Self-reported behavior | (7-point)  Once a day or more (7)  Once a week (6)  Once a month (5)  Once every 2-3 months (4)  Once every six months (3)  Once a year (2)  Never (1) |
|  |  | Q7_b | Intervene in an incident of sexual harassment in a field setting | H2a |  |  |
|  |  | Q7_c | Report an incident of sexual harassment that took place a field setting | H2a |  |  |
|  |  | Q7_d | Create or help create resources (e.g. policies, codes of conduct) about how to prevent, intervene in, or report sexual harassment in a field setting | H2a |  |  |
|  |  | Q7_e | Encourage someone else to take action to prevent sexual harassment in field settings (for example, you might encourage others to seek out resources, intervene in an incident, or report an incident) | H2a |  |  |
| 1,3 | Q8 While you are working in the field for CDFW, approximately how frequently do you do the follow actions in response to**sexual assault?** | Q8_a | Seek out resources about how to prevent, intervene in, or report sexual assault in a field setting | H2a | Self-reported behaviour | (7-point)  Once a day or more (7)  Once a week (6)  Once a month (5)  Once every 2-3 months (4)  Once every six months (3)  Once a year (2)  Never (1) |
|  |  | Q8_b | Intervene in an incident of sexual assault in a field setting | H2a |  |  |
|  |  | Q8_c | Report an incident of sexual assault that took place a field setting | H2a |  |  |
|  |  | Q8_d | Create or help create resources (e.g. policies, codes of conduct) about how to prevent, intervene in, or report sexual assault in a field setting | H2a |  |  |
|  |  | Q8_e | Encourage someone else to take action to prevent sexual assault in field settings (for example, you might encourage others to seek out resources, intervene in an incident, or report an incident) | H2a |  |  |
| 1,3 |  | Q9 | Have you ever taken any other action to prevent sexual harassment and/or assault in field settings? | H2a | Self-reported behavior | Open-ended |
| 1,3 |  | Q10 | Approximately how many people have you encouraged to take action to prevent sexual harassment and/or sexual assault in field settings? | H2a | Self-reported behavior | (5-point)  -Most or all of the people I work with (5)  -About half of the people I work with (4)  -Some people I work with (3)  -One or two people I work with (2)  -None (1) |
| 1,2,3 | Q11. In the next six months, how likely are you to do the following in response to sexual harassment? | Q11_a | Seek out resources about how to prevent, intervene in, or report sexual harassment in a field setting | H2b | Behavioral intention | (7-point)  -Very likely (7)  -Likely (6)  -Somewhat likely (5)  -Neither likely nor unlikely (4)  -Somewhat unlikely (3)  -Unlikely (2)  -Very unlikely (1) |
|  |  | Q11_b | Intervene in an incident of sexual harassment if I see it occur in a field setting | H2b |  |  |
|  |  | Q11_c | Report an incident of sexual harassment if I see it occur in a field setting | H2b |  |  |
|  |  | Q11_d | Encourage others to take action to prevent sexual harassment in field settings. | H2b |  |  |
| 1,2,3 | Q12 In the next six months, how likely are you to do the following in response to sexual assault? | Q12_a | Seek out resources about how to prevent, intervene in, or report sexual assault in a field setting | H2b | Behavioral intention | (7-point)  -Very likely (7)  -Likely (6)  -Somewhat likely (5)  -Neither likely nor unlikely (4)  -Somewhat unlikely (3)  -Unlikely (2)  -Very unlikely (1) |
|  |  | Q12_b | Intervene in an incident of sexual assault if I see it occur in a field setting | H2b |  |  |
|  |  | Q12_c | Report an incident of sexual assault if I see it occur in a field setting | H2b |  |  |
|  |  | Q12_d | Encourage others to take action to prevent sexual assault in field settings. | H2b |  |  |
| 2 |  | Q13 | If you were to take action to prevent sexual harassment and/or sexual assault in field settings, what would be your primary reason for doing so? |  |  | Open-ended |
| 2 |  | Q14 | What concerns would make you hesitate to take action to prevent sexual harassment and/or sexual assault in field settings? |  |  | Open-ended |
| 2 |  | Q16 | Would you say your general attitude towards sexual harassment and assault prevention training is positive, negative, or neutral? |  |  | (7-point)  -Extremely positive (7)  -Moderately positive (6)  -Slightly positive (5)  -Neutral (4)  -Slightly negative (3)  -Moderately negative (2)  -Extremely negative (1) |
| 2 |  | Q17 | Please rate the overall quality of the facilitator-led lecture portion of the workshop. |  |  |  |
| 2 |  | Q18 | Please rate the overall quality of the scenario-based breakout discussion portion of the workshop. |  |  |  |
| 2 |  | Q19 | How likely or unlikely are you to incorporate the content of this workshop into your field setting? |  |  | (7-point)  -Very likely (7)  -Likely (6)  -Somewhat likely (5)  -Neither likely nor unlikely (4)  -Somewhat unlikely (3)  -Unlikely (2)  -Very unlikely (1) |
| 2 |  | Q20 | How likely are you to recommend this workshop to a friend or colleague? |  |  | (7-point)  -Very likely (7)  -Likely (6)  -Somewhat likely (5)  -Neither likely nor unlikely (4)  -Somewhat unlikely (3)  -Unlikely (2)  -Very unlikely (1) |
| 2 |  | Q21 | What did you find more interesting or useful about this workshop? |  |  | Open-ended |
| 2 |  | Q22 | Do you have any other suggestions for what we could do to improve this workshop in the future? |  |  | Open-ended |
| 1 |  |  | What is your current classification? |  | Position |  |
|  |  |  | How long have you been employed in your current or any position with CDFW? |  | Tenure |  |
|  |  |  | What is your gender? |  | Gender |  |
|  |  |  | Do you identify as transgender? |  |  |  |
|  |  |  | What is your age (in years)? |  | Age |  |
|  |  |  | What is your highest level of education? |  | Educational background |  |
|  |  |  | What is your race and ethnicity? (Please check all that apply.) |  | Race and ethnicity |  |

**Table S2.** Survey instruments to be used in this study and timing of distribution to participants.

| **Phase** | **Description** | **Purpose** |
| --- | --- | --- |
| Time 1 | Pre-training administered to CDFW staff **prior to** participating in the BBFF training. | Compare matched responses before and after participating in the training |
| Time 2 | Post-training administered to CDFW staff **immediately after** participating in the BBFF training. | Compare matched responses before and after participating in the training |
| Time 3 | Midpoint survey distributed to all CDFW participants **halfway through** all trainings are complete. For participants in the treatment group this was administered 1-2 months after receiving training; for participants in the control group this wan administered 1-2 months after completion of first survey. | Provide control group (untrained staff) with which to compare treatment group (trained staff) |

#

#

**Table S3.** Survey response data among three survey waves.

| **Time Period** | **Time 1 N (%)**  (Pre-training survey) | **Time 2 N (%)**  (Post-training survey) | **Time 3 N (%)**  (Midpoint treatment-control data) | **Total N (%)** |
| --- | --- | --- | --- | --- |
| **Total # of Surveys Completed** | 458 (100) | 342 (100) | 248 (100) | 1,048 (100) |
| **Age Median (IQR) (Min-Max)** | 35 (20) (21-81) | 43 (18) (25-66) | 43 (21) (25-66) | 38 (21) (21-81) |
| **Age Categories** |  |  |  |  |
| 21-29 | 123 (32.1) | 4 (8.7) | 7 (11.1) | 134 (27.2) |
| 30-49 | 171 (44.7) | 25 (54.4) | 34 (53.9) | 230 (46.8) |
| 50-64 | 82 (21.4) | 15 (32.6) | 20 (31.8) | 117 (23.8) |
| 65+ | 7 (1.8) | 2 (4.4) | 2 (3.2) | 11 (2.2) |
| Missing | 75 | 296 | 185 | 556 |
| **Gender** |  |  |  |  |
| Female | 200 (49.3) | 29 (60.4) | 34 (53.9) | 263 (50.9) |
| Male | 201 (49.5) | 18 (37.5) | 27 (42.9) | 246 (47.6) |
| Other (Non-binary, prefer not to state) | 5 (1.2) | 1 (2.1) | 2 (3.2) | 8 (1.5) |
| Missing | 52 | 294 | 185 | 531 |
| **Race** |  |  |  |  |
| White | 248 (63.8) | 28 (60.9) | 37 (58.7) | 313 (62.8) |
| Underrepresented Minority | 141 (36.3) | 18 (39.3) | 26 (41.3) | 185 (37.2) |
| Missing | 69 | 296 | 185 | 550 |
| **Level of Education** |  |  |  |  |
| High school graduate (or equivalent) | 5 (1.2) | 1 (2.1) | 1 (1.6) | 7 (1.4) |
| Some college or Associate’s degree | 39 (9.6) | 8 (16.7) | 11 (17.5) | 58 (11.2) |
| Bachelor’s degree | 256 (62.9) | 25 (52.1) | 30 (47.7) | 311 (60.0) |
| Graduate degree or higher | 107 (26.3) | 14 (29.2) | 21 (33.3) | 142 (27.4) |
| Missing | 51 | 294 | 185 | 530 |
| **Experience** |  |  |  |  |
| Less than 6 months | 47 (11.5) | 3 (6.3) | 2 (3.2) | 52 (10.0) |
| 6 months to a year | 60 (14.7) | 5 (10.4) | 5 (7.9) | 70 (13.5) |
| 1-2 years | 43 (10.5) | 5 (10.4) | 6 (9.5) | 54 (10.4) |
| 2-5 years | 91 (22.3) | 8 (16.7) | 15 (23.8) | 114 (22.0) |
| 5 -10 years | 53 (12.9) | 10 (20.8) | 11 (17.5) | 74 (14.2) |
| >10 years | 115 (28.1) | 17 (35.4) | 24 (38.1) | 156 (30.0) |
| Missing | 49 | 294 | 185 | 528 |
| **Position** |  |  |  |  |
| Administrative Officer | 6 (1.5) | 1 (2.1) | 2 (3.2) | 9 (1.7) |
| Environmental Program Manager | 11 (2.7) | 0 (0.0) | 3 (4.8) | 14 (2.7) |
| Environmental Scientist | 84 (20.6) | 12 (25.0) | 12 (19.1) | 108 (20.8) |
| Fish Hatchery Manager | 4 (0.9) | 0 (0.0) | 0 (0.0) | 4 (0.8) |
| Research Scientist Supervisor | 2 (0.5) | 0 (0.0) | 0 (0.0) | 2 (0.4) |
| Scientific Aide | 146 (35.8) | 4 (8.3) | 7 (11.1) | 157 (30.3) |
| Senior Environmental Program Manager | 1 (0.3) | 0 (0.0) | 0 (0.0) | 14 (2.7) |
| Senior Environmental Scientist | 80 (19.6) | 4 (8.3) | 8 (12.7) | 92 (17.7) |
| Other (please specify): | 74 (18.1) | 27 (56.30 | 31 (49.2) | 132 (25.4) |
| Missing | 50 | 294 | 185 | 529 |

**Table S4.** Results of linear models testing effect on demographic and occupational groups on change in scores related to knowledge, self-efficacy, and behavioral intention (n=196).

| **Concept** | **Variable** | **Term** | **β** | **CI lower** | **CI upper** | **Std. error** | **p-value** | **Sig.** |
| --- | --- | --- | --- | --- | --- | --- | --- | --- |
| Knowledge | Knowledge | (Intercept) | 1.364 | -2.037 | 4.765 | 1.690 | 0.424 |  |
|  |  | gender:Woman | 1.747 | 0.733 | 2.761 | 0.504 | 0.001 | ** |
|  |  | race:white | 0.093 | -1.033 | 1.220 | 0.560 | 0.869 |  |
|  |  | positionScientific Aide | 0.769 | -1.683 | 3.221 | 1.218 | 0.531 |  |
|  |  | positionScientist | 0.826 | -1.943 | 3.594 | 1.375 | 0.551 |  |
|  |  | positionSenior scientist | -0.457 | -2.825 | 1.911 | 1.176 | 0.699 |  |
|  |  | age_21-29 | -2.158 | -5.425 | 1.109 | 1.623 | 0.190 |  |
|  |  | age_30_49 | -0.830 | -3.838 | 2.177 | 1.494 | 0.581 |  |
|  |  | age_50_64 | -0.156 | -3.083 | 2.771 | 1.454 | 0.915 |  |
|  |  | age_65_79 | -0.932 | -4.453 | 2.588 | 1.749 | 0.597 |  |
| Self-efficacy | Preventing | (Intercept) | 0.819 | -2.293 | 3.931 | 1.546 | 0.599 |  |
|  |  | gender:Woman | 0.940 | 0.011 | 1.868 | 0.461 | 0.047 |  |
|  |  | race:white | -0.326 | -1.357 | 0.704 | 0.512 | 0.527 |  |
|  |  | positionScientific Aide | 1.428 | -0.816 | 3.672 | 1.115 | 0.207 |  |
|  |  | positionScientist | 0.966 | -1.567 | 3.500 | 1.258 | 0.446 |  |
|  |  | positionSenior scientist | 0.008 | -2.159 | 2.175 | 1.077 | 0.994 |  |
|  |  | age_21-29 | -2.167 | -5.157 | 0.822 | 1.485 | 0.151 |  |
|  |  | age_30_49 | -0.898 | -3.651 | 1.854 | 1.367 | 0.514 |  |
|  |  | age_50_64 | 0.008 | -2.671 | 2.686 | 1.331 | 0.995 |  |
|  |  | age_65_79 | -0.659 | -3.881 | 2.562 | 1.600 | 0.682 |  |
|  | Intervening | (Intercept) | 1.149 | -1.009 | 3.307 | 1.072 | 0.289 |  |
|  |  | gender:Woman | 0.765 | 0.121 | 1.408 | 0.320 | 0.021 | * |
|  |  | race:white | -0.269 | -0.984 | 0.445 | 0.355 | 0.452 |  |
|  |  | positionScientific Aide | 0.866 | -0.690 | 2.422 | 0.773 | 0.268 |  |
|  |  | positionScientist | 0.742 | -1.014 | 2.499 | 0.873 | 0.400 |  |
|  |  | positionSenior scientist | 0.120 | -1.382 | 1.623 | 0.746 | 0.873 |  |
|  |  | age_21-29 | -1.788 | -3.861 | 0.285 | 1.030 | 0.089 |  |
|  |  | age_30_49 | -1.186 | -3.095 | 0.722 | 0.948 | 0.217 |  |
|  |  | age_50_64 | -0.721 | -2.578 | 1.137 | 0.923 | 0.439 |  |
|  |  | age_65_79 | -1.074 | -3.308 | 1.159 | 1.110 | 0.338 |  |
|  | Reporting | (Intercept) | 0.894 | -1.609 | 3.398 | 1.244 | 0.476 |  |
|  |  | gender:Woman | 0.747 | 0.000 | 1.493 | 0.371 | 0.050 | * |
|  |  | race:white | 0.155 | -0.674 | 0.984 | 0.412 | 0.708 |  |
|  |  | positionScientific Aide | 0.721 | -1.084 | 2.525 | 0.897 | 0.426 |  |
|  |  | positionScientist | -0.203 | -2.241 | 1.834 | 1.012 | 0.842 |  |
|  |  | positionSenior scientist | -0.050 | -1.793 | 1.693 | 0.866 | 0.954 |  |
|  |  | age_21-29 | -1.577 | -3.982 | 0.828 | 1.195 | 0.193 |  |
|  |  | age_30_49 | -0.993 | -3.207 | 1.221 | 1.100 | 0.371 |  |
|  |  | age_50_64 | 0.094 | -2.060 | 2.249 | 1.070 | 0.930 |  |
|  |  | age_65_79 | -0.697 | -3.289 | 1.894 | 1.287 | 0.591 |  |
|  | Encouraging others | (Intercept) | 1.035 | -1.425 | 3.494 | 1.222 | 0.401 |  |
|  |  | gender:Woman | 0.868 | 0.135 | 1.602 | 0.364 | 0.021 | * |
|  |  | race:white | -0.080 | -0.894 | 0.735 | 0.405 | 0.845 |  |
|  |  | positionScientific Aide | 0.399 | -1.375 | 2.172 | 0.881 | 0.653 |  |
|  |  | positionScientist | 0.145 | -1.857 | 2.147 | 0.995 | 0.885 |  |
|  |  | positionSenior scientist | 0.045 | -1.668 | 1.757 | 0.851 | 0.958 |  |
|  |  | age_21-29 | -1.496 | -3.859 | 0.867 | 1.174 | 0.209 |  |
|  |  | age_30_49 | -1.168 | -3.344 | 1.007 | 1.081 | 0.285 |  |
|  |  | age_50_64 | -0.234 | -2.351 | 1.883 | 1.052 | 0.825 |  |
|  |  | age_65_79 | -0.767 | -3.313 | 1.779 | 1.265 | 0.547 |  |
| Behavioral intention | Seeking resources | (Intercept) | 1.961 | -3.354 | 7.276 | 2.641 | 0.462 |  |
|  |  | gender:Woman | 0.497 | -1.089 | 2.082 | 0.787 | 0.531 |  |
|  |  | race:white | 0.952 | -0.809 | 2.712 | 0.875 | 0.282 |  |
|  |  | positionScientific Aide | -1.561 | -5.393 | 2.271 | 1.904 | 0.416 |  |
|  |  | positionScientist | -1.158 | -5.484 | 3.168 | 2.149 | 0.593 |  |
|  |  | positionSenior scientist | -0.912 | -4.613 | 2.789 | 1.839 | 0.622 |  |
|  |  | age_21-29 | -0.755 | -5.860 | 4.351 | 2.537 | 0.767 |  |
|  |  | age_30_49 | -1.451 | -6.151 | 3.250 | 2.335 | 0.537 |  |
|  |  | age_50_64 | -0.062 | -4.637 | 4.513 | 2.273 | 0.978 |  |
|  |  | age_65_79 | -0.980 | -6.482 | 4.522 | 2.733 | 0.721 |  |
|  | Intervening | (Intercept) | 2.203 | -2.079 | 6.485 | 2.127 | 0.306 |  |
|  |  | gender:Woman | 0.353 | -0.924 | 1.630 | 0.634 | 0.580 |  |
|  |  | race:white | 1.028 | -0.390 | 2.446 | 0.705 | 0.151 |  |
|  |  | positionScientific Aide | -1.483 | -4.570 | 1.604 | 1.534 | 0.339 |  |
|  |  | positionScientist | -1.281 | -4.766 | 2.205 | 1.732 | 0.463 |  |
|  |  | positionSenior scientist | -1.730 | -4.712 | 1.251 | 1.481 | 0.249 |  |
|  |  | age_21-29 | -0.943 | -5.056 | 3.171 | 2.043 | 0.647 |  |
|  |  | age_30_49 | -2.003 | -5.790 | 1.784 | 1.881 | 0.293 |  |
|  |  | age_50_64 | 0.518 | -3.167 | 4.204 | 1.831 | 0.778 |  |
|  |  | age_65_79 | 0.399 | -4.034 | 4.831 | 2.202 | 0.857 |  |
|  | Reporting | (Intercept) | 1.149 | -3.008 | 5.305 | 2.065 | 0.581 |  |
|  |  | gender:Woman | 0.105 | -1.134 | 1.345 | 0.616 | 0.865 |  |
|  |  | race:white | 0.776 | -0.601 | 2.152 | 0.684 | 0.263 |  |
|  |  | positionScientific Aide | 0.548 | -2.449 | 3.544 | 1.489 | 0.715 |  |
|  |  | positionScientist | -0.994 | -4.377 | 2.390 | 1.681 | 0.557 |  |
|  |  | positionSenior scientist | -0.424 | -3.318 | 2.470 | 1.438 | 0.769 |  |
|  |  | age_21-29 | -1.562 | -5.555 | 2.431 | 1.984 | 0.435 |  |
|  |  | age_30_49 | -1.436 | -5.112 | 2.240 | 1.826 | 0.436 |  |
|  |  | age_50_64 | 0.237 | -3.341 | 3.814 | 1.777 | 0.895 |  |
|  |  | age_65_79 | -1.074 | -5.377 | 3.228 | 2.137 | 0.618 |  |
|  | Encouraging others | (Intercept) | 1.706 | -2.154 | 5.566 | 1.918 | 0.378 |  |
|  |  | gender:Woman | 0.085 | -1.066 | 1.236 | 0.572 | 0.882 |  |
|  |  | race:white | 1.191 | -0.088 | 2.469 | 0.635 | 0.067 |  |
|  |  | positionScientific Aide | -1.309 | -4.092 | 1.474 | 1.383 | 0.349 |  |
|  |  | positionScientist | -0.699 | -3.841 | 2.443 | 1.561 | 0.656 |  |
|  |  | positionSenior scientist | -1.397 | -4.084 | 1.291 | 1.335 | 0.301 |  |
|  |  | age_21-29 | -0.491 | -4.199 | 3.217 | 1.842 | 0.791 |  |
|  |  | age_30_49 | -1.882 | -5.296 | 1.531 | 1.696 | 0.273 |  |
|  |  | age_50_64 | 0.989 | -2.333 | 4.312 | 1.651 | 0.552 |  |
|  |  | age_65_79 | -0.353 | -4.349 | 3.643 | 1.985 | 0.860 |  |

**​​**

**Table S5.** Results of linear models testing effect on personal norms and self-reported prevention behavior on composite change in scores related to behavioral intention.

| **Predictor** | **Behavioral intention variable** | **Term** | **β** | **CI lower** | **CI upper** | **Std. error** | **p-value** | **Sig.** |
| --- | --- | --- | --- | --- | --- | --- | --- | --- |
| Prevention behavior | Seeking resources | (Intercept) | 0.669 | -0.330 | 0.859 | 0.247 | 0.381 |  |
|  |  | behavior_groupLow | 0.264 | -0.330 | 0.859 | 0.301 | 0.381 |  |
|  | Intervening | (Intercept) | 0.941 | -0.851 | 0.325 | 0.244 | 0.379 |  |
|  |  | behavior_groupLow | -0.263 | -0.851 | 0.325 | 0.298 | 0.379 |  |
|  | Reporting | (Intercept) | 0.856 | -0.701 | 0.460 | 0.241 | 0.683 |  |
|  |  | behavior_groupLow | -0.120 | -0.701 | 0.460 | 0.294 | 0.683 |  |
|  | Encouraging others | (Intercept) | 0.814 | -0.661 | 0.455 | 0.232 | 0.716 |  |
|  |  | behavior_groupLow | -0.103 | -0.661 | 0.455 | 0.283 | 0.716 |  |
| Personal norms | Seeking resources | (Intercept) | 0.867 | -0.532 | 0.605 | 0.192 | 0.899 |  |
|  |  | norm_groupLow | 0.036 | -0.532 | 0.605 | 0.288 | 0.899 |  |
|  | Intervening | (Intercept) | 0.878 | -0.778 | 0.356 | 0.191 | 0.464 |  |
|  |  | norm_groupLow | -0.211 | -0.778 | 0.356 | 0.287 | 0.464 |  |
|  | Reporting | (Intercept) | 0.878 | -0.752 | 0.368 | 0.189 | 0.500 |  |
|  |  | norm_groupLow | -0.192 | -0.752 | 0.368 | 0.284 | 0.500 |  |
|  | Encouraging others | (Intercept) | 0.939 | -0.908 | 0.158 | 0.180 | 0.167 |  |
|  |  | norm_groupLow | -0.375 | -0.908 | 0.158 | 0.270 | 0.167 |  |

**Table S6.** Results of linear regressions testing effect on pre- and post-training knowledge, self-efficacy, and behavioral intention without controlling for race, gender or any other demographic variables (n=196).

| **Concept** | **Variable** | **Term** | **β** | **CI lower** | **CI upper** | **Std. error** | **p-value** | **Sig.** |
| --- | --- | --- | --- | --- | --- | --- | --- | --- |
| Knowledge | Knowledge | (Intercept) | 4.81 | 4.623 | 4.999 | 0.10 | <0.001 | *** |
|  |  | post-training | 1.17 | 0.902 | 1.429 | 0.13 | <0.001 | *** |
| Self-efficacy | Preventing | (Intercept) | 5.16 | 4.980 | 5.345 | 0.09 | <0.001 | *** |
|  |  | post-training | 0.60 | 0.341 | 0.853 | 0.13 | <0.001 | *** |
|  | Intervening | (Intercept) | 5.53 | 5.359 | 5.695 | 0.09 | <0.001 | *** |
|  |  | post-training | 0.55 | 0.316 | 0.787 | 0.12 | <0.001 | *** |
|  | Reporting | (Intercept) | 5.71 | 5.560 | 5.867 | 0.08 | <0.001 | *** |
|  |  | post-training | 0.59 | 0.373 | 0.803 | 0.11 | <0.001 | *** |
|  | Encouraging others | (Intercept) | 5.58 | 5.409 | 5.748 | 0.09 | <0.001 | *** |
|  |  | post-training | 0.53 | 0.291 | 0.767 | 0.12 | <0.001 | *** |
| Behavioral intention | Seeking resources | (Intercept) | 4.30 | 4.023 | 4.567 | 0.14 | <0.001 | *** |
|  |  | post-training | 0.87 | 0.489 | 1.249 | 0.19 | <0.001 | *** |
|  | Intervening | (Intercept) | 5.34 | 5.088 | 5.595 | 0.13 | <0.001 | *** |
|  |  | post-training | 0.74 | 0.387 | 1.096 | 0.18 | <0.001 | *** |
|  | Reporting | (Intercept) | 5.44 | 5.190 | 5.695 | 0.13 | <0.001 | *** |
|  |  | post-training | 0.74 | 0.392 | 1.098 | 0.18 | <0.001 | *** |
|  | Encouraging others | (Intercept) | 5.15 | 4.901 | 5.400 | 0.13 | <0.001 | *** |
|  |  | post-training | 0.72 | 0.376 | 1.073 | 0.18 | <0.001 | *** |
